# Characterization and Early Response of the DEAD Gene Family to Heat Stress in Tomato

**DOI:** 10.3390/plants14081172

**Published:** 2025-04-09

**Authors:** Yanyan Yan, Chao Yu, Bolun Xie, Hui Zhou, Caiyu Zhang, Li Tian

**Affiliations:** 1 Collaborative Innovation Center for Efficient and Green Production of Agriculture in Mountainous Areas of Zhejiang Province, College of Horticulture Science, Zhejiang A&F University, Hangzhou 311300, China; yanyan@zafu.edu.cn (Y.Y.); yuchaocc0401@163.com (C.Y.); 2023614021057@stu.zafu.edu.cn (B.X.); 2023614022082@stu.zafu.edu.cn (H.Z.); 2 Key Laboratory of Quality and Safety Control for Subtropical Fruit and Vegetable, Ministry of Agriculture and Rural Affairs, Zhejiang A&F University, Hangzhou 311300, China; 3 Institute of Agricultural Experiment Station of Changxing Substation, Zhejiang University, Hangzhou 310058, China; caiyuzh@126.com

**Keywords:** tomato, *SlDEAD*, subcellular localization, high temperature

## Abstract

The DEAD-box RNA helicase family, acting as a critical regulator in RNA metabolism, plays a vital role in plant growth, development, and adaptation to various stresses. Although a number of DEAD proteins have been reported to participate in heat stress response in several species, the response of DEAD-box RNA helicases to heat stress has not been comprehensively analyzed in tomato. In this study, 42 *SlDEAD* genes were identified from the tomato genome. Evolutionary analysis of *DEAD* family genes across different plant species reveals that *DEAD* family genes can be segregated into five groups. A comprehensive analysis of their physicochemical properties, gene structure, chromosome location, and conserved motifs unveils diversity among the members of the *SlDEAD* family. An investigation into the subcellular localization of seven SlDEAD proteins indicates that SlDEAD7, SlDEAD14, and SlDEAD26 are located in the endoplasmic reticulum, and SlDEAD40 is located in the endoplasmic reticulum and nucleus, whereas SlDEAD17, SlDEAD25, and SlDEAD35 are located in the chloroplast. The expression of 37 out of 42 *SlDEAD* genes was responsive to heat stress induction. During the early stage of high-temperature treatment, they exhibited five distinct expression patterns. These findings contribute to a deeper comprehension of the evolution, expansion complexity, and function of *SlDEAD* genes and provide insights into the potential role of *SlDEAD* genes in tomato tolerance to heat stress.

## 1. Introduction

Tomato is a globally cultivated vegetable crop. It not only serves as a staple ingredient in diverse cuisines worldwide but also significantly contributes to farmers’ incomes and the stability of the global vegetable supply. Global warming leads to an increase in annual temperature. Severe high-temperature stress can induce irreversible damage to plant growth, which may pervade the entire life cycle of plants [1]. Although tomato is considered a thermophilic crop, high-temperature stress can markedly affect its productivity through multiple mechanisms. High temperatures lead to alterations in the fluidity of the thylakoid membrane of chloroplasts, thereby reducing the maximum photochemical efficiency (Fv/Fm). When the temperature exceeds 35 °C, the activity of the Rubisco enzyme is inhibited, leading to a decline in the net photosynthetic rate [2]. Moreover, when pollen mother cells experience high temperatures during the meiosis stage, the abnormal programmed cell death of tapetal cells occurs. This results in over 70% pollen abortion and a 35–60% reduction in fruit setting rates [3]. Additionally, high temperatures can trigger an outburst of reactive oxygen species, shorten leaf lifespan, and reduce root activity. As a consequence, the supply of carbon assimilated during the fruit expansion period is reduced [4]. Therefore, high-temperature stress significantly impairs the growth and productivity of tomatoes. Understanding the molecular mechanism underlying the impact of high-temperature stress on tomato cultivation is consequently of critical significance. This understanding is essential for developing effective mitigation strategies and breeding heat-tolerant tomato varieties, which ensures tomato productivity and, in turn, global food security and the agricultural economy.

At the molecular level, high temperatures have profound impacts on gene expression, particularly at transcriptional and post-transcriptional levels. Transcription factors play a central role in the transcription process. For instance, heat shock transcription factors (HSFs) are usually activated upon exposure to high temperatures [1]. They bind to specific DNA sequences called heat shock elements (HSEs) located in the promoter regions of heat-responsive genes. This binding event initiates the transcription of genes encoding heat shock proteins (HSPs) and other stress-related proteins [5]. HSPs act as molecular chaperones, helping to refold misfolded proteins and prevent protein aggregation, which is a common consequence of high-temperature-induced cellular stress. In addition to HSFs, other transcription factors, such as dehydration–responsive element–binding proteins (DREBs), are also involved in the heat stress response [6]. DREBs can bind to dehydration–responsive elements (DREs) in the promoter regions of target genes. Some of these target genes are associated with enhancing plant tolerance to heat stress by regulating various physiological and biochemical processes [7]. These transcription factors and their associated heat-responsive genes have been employed in genetic engineering and marker-assisted selection techniques, offering targeted strategies to enhance heat tolerance in tomatoes. For example, four tomato HSF genes were reported to be upregulated under high-temperature treatment, and their expression levels were closely linked to the thermophenotypes of eight tomato genotypes [8]. This finding indicates that the identified HSF genes can be utilized to assist in screening for potential thermotolerant cultivars.

At the post-transcriptional level, RNA-binding proteins emerge as pivotal regulators. RBPs possess the ability to specifically recognize and bind to target RNAs. Through this binding interaction, they orchestrate a series of crucial processes, including RNA processing and mRNA transport, stability, and translation [9,10]. Under heat stress conditions, the regulatory functions executed by RBPs play a vital role in modulating gene expression at the RNA level [11,12], ensuring the continuous and appropriate expression of heat-responsive genes. For example, mutations of the ESR1 (enhanced stress response 1) gene, which encodes an *Arabidopsis* K Homology (KH) Domain containing RBPs, confer increased heat tolerance [13]. This increased tolerance is achieved by altering the expression of several abiotic stimuli genes, such as defensin-like family genes.

RNA helicases (RHs), the highly conserved proteins in all kingdoms of life [14,15], constitute a large group of enzymes that primarily function in the unwinding of RNA molecules. They are widely recognized for their involvement in the remodeling of RNA and ribonucleoprotein complexes (RNPs), which is mainly dependent on their binding activity to RNAs in an ATP-dependent reaction [16]. The RH family genes have been progressively identified at the genomic level in model crops. Specifically, there are 115 RH family genes in rice [17], 113 in *Arabidopsis* [17], 136 in maize [18], 213 in soybean [18], and 161 in cotton [19].

Based on protein sequence homology and their oligomeric state, RHs are divided into six superfamilies: SF1 to SF6. Among them, members from SF3 to SF6 usually function in ring-shaped hexametric toroid structures [20]. In contrast, helicases from SF1 and SF2 are typically non-oligomeric proteins containing a core structure composed of two RecA-like domains [21]. In eukaryotes, the majority of RNA helicases belong to the SF2 superfamily, with only a small fraction being SF1 proteins. According to the conserved motif and structural or mechanistic features, RHs in the SF2 superfamily are further divided into several subfamilies [22,23,24]. Among these subfamilies, DEAD (Asp-Glu-Ala-Asp)-box-containing RHs are the largest group ever discovered [21].

Due to their activities in RNA remodeling, DEAD-box RHs play specific roles in almost all aspects of RNA processing and metabolism, including double-stranded RNA unwinding, pre-mRNA processing, and RNA export, translation, storage, and decay in eukaryotes [25,26,27]. In recent years, the mechanism by which DEAD-box RNA helicases regulate RNA transcription and post-transcription has been gradually elucidated, and growing evidence indicates that DEAD-box RHs are essential for organelle biogenesis, plant growth, development, and stress resistance [28,29,30,31,32,33,34,35,36], particularly in relation to temperature stresses. For example, *Arabidopsis* DEAD-box RH AtRH7/PRH75 was involved in the processing of 18S pre-rRNA and ribosome assembly, which resulted in auxin-related developmental defects and cold sensitivity in *AtRH7*/*PRH75*-knockout mutants [37]. The cold-induced DEAD-box RH AtRCF1 is required for the correct splicing of the pre-mRNA of cold-responsive genes like *CIR1*, *SPFH*, *PRR5*, and *SK12*, which contributed to the enhanced tolerance of *Arabidopsis* to low temperature and freezing stresses [25]. In rice, the mutation of the gene *TCD33* that encodes a chloroplast-located DEAD-box RH induced an albino phenotype and severe defects in the chloroplast structure under low-temperature conditions [38]. Rice OsRH42 was reported to interact with U2 small nuclear RNA and was located in splicing spots in the nucleus [33], and its knockout caused defects in pre-mRNA splicing and plant growth under cold stress. Collectively, these studies imply that DEAD-box RHs play crucial roles in plant tolerance to extreme temperatures.

Although the function of DEAD-box RHs has been extensively explored in *Arabidopsis* and rice, only a few studies on DEAD-box RHs in tomato have been reported. A tomato mutant, designated as *res* (*restored cell structure by salinity*), was named in accordance with its phenotype, wherein the morphological alterations and cellular disorganization under normal conditions were restored under salt stress conditions [39]. A subsequent study on the *res* mutant disclosed that the *res* gene encodes a chloroplast-targeted *DEAD-box RNA helicase 39* (SlDEAD39) [40,41]. SlDEAD39 modulated the maturation of chloroplast 23S rRNA by binding to the RNA molecules containing the hidden break-B site [41], which might contribute to its roles in tomato plant development and salt tolerance. In addition, *SlDEAD23* and *SlDEAD35* were reported to potentially be involved in abiotic stress, such as salt and cold, as well as the biotic stress of ToLCNDV infection [29]. Moreover, *SlDEAD31* was proven to positively regulate tomato tolerance to salt and drought stresses, potentially via the upregulation of stress-related genes [42]. However, the response of tomato DEAD-box RHs to heat stress has not been deeply analyzed.

In this study, we conducted a comprehensive analysis of the characteristics and subcellular localization of 42 DEAD-box RHs in tomato and investigated their responses to heat stress. We found that 42 SlDEAD proteins exhibited diverse patterns in both their subcellular localization and expression in response to heat stress. These findings provide new clues for the future study of the functions of SlDEAD in tomato and to identify potential target genes for molecular breeding aimed at enhancing the heat tolerance of tomatoes.

## 2. Results

### 2.1. Identification and Phylogenetic Analysis of SlDEAD Proteins

A total of 42 *SlDEAD* genes, namely, *SlDEAD1* to *SlDEAD42*, as named in a previous study [43], were identified from the tomato genome. Their gene ID and the corresponding homologs in *Arabidopsis* are presented in Table 1. The amino acid sequence of 42 SlDEAD proteins varies in length, ranging from 394 to 1221 amino acids. The smallest SlDEAD protein is SlDEAD41, which has a molecular weight of 44.85 KDa. In contrast, the largest one is SlDEAD2, comprising 1221 amino acid residues with a molecular weight of 135.06 KDa (Table 1). The isoelectric points of SlDEAD proteins span from 5.21 to 9.96, and approximately one-third of them possess an isoelectric point of less than 7. The instability indexes range from 33.14 to 63.63. Only 12 of them have an instability index below 40, indicating that the majority of these proteins may be unstable.

**Figure 1:**
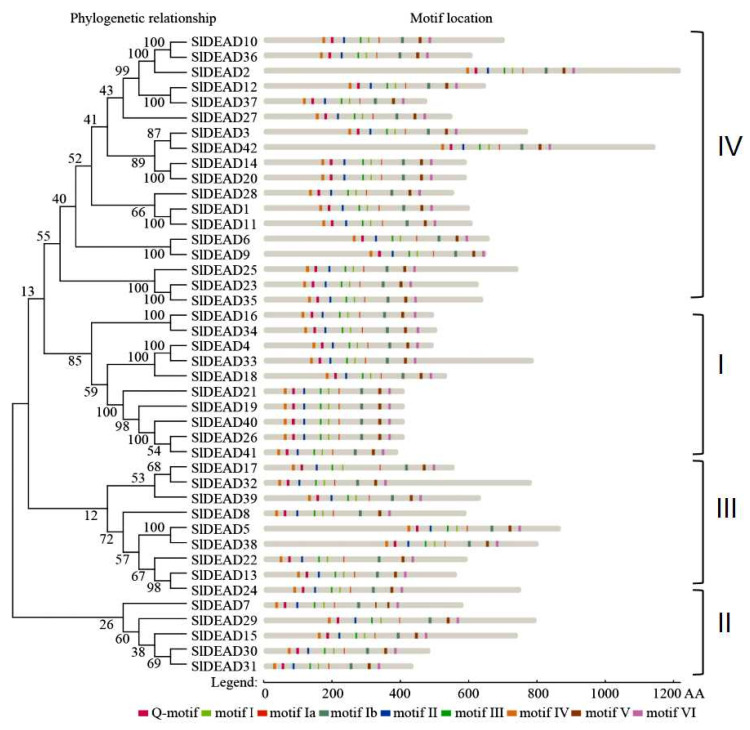
Phylogenetic tree and motif analysis of SlDEAD family. The evolutionary relationships among the SlDEAD proteins in tomato are presented on the left, and the classified groups are annotated on the right. The conserved motifs are depicted as colored boxes. The conserved amino acid sequence within each motif are gaccpohlQ in Q-motif, SxtGoGKt in motif I, PtreLk in motif Ia, TPmkl in motif Ib, DEAD in motif II, lisAT in motif III, llfhxq+cx in motif IV, Tdvu-bGld in motif V, and HR*GRsmR in motif VI. The symbols used in the animo acid sequence represent different combinations of amino acids, in which O is for S, T, M, or F; l is for I, L, V, or M; x is for any residue; a is for F, W, Y, M, L, or I; c is for D, E, H, K, or R; h is for A, F, G, I, L, M, P, V, W, Y, T, C, E, K, S, Q, or D; + is for H, K, R, V, I, L, C, Q, or A; u is for A, V, L, F, or S; v is for A, E, L, V, or I; d is for D, S, or N; b is for R or K; f is for F or Y; − is for A, V, S, G, or T; * is for V, I, S, T, or L; m is for A or G; + is for V, I, L, C, K, R, Q, A, or H; q is for T, S, N, R, Y, or K; k for A, C, R, T, or S; e is for M or E; t is for T, N, or S; and r is for R or V.

Sequence alignment and phylogenetic analysis reveal that these SlDEAD proteins are clustered into four clades (Figure 1). Clade I contains only five SlDEAD proteins, including SlDEAD7, SlDEAD15, SlDEAD29, SlDEAD30, and SlDEAD31. Clade IV comprises 18 SlDEAD proteins—SlDEAD1, SlDEAD2, SlDEAD3, SlDEAD6, SlDEAD9, SlDEAD10, SlDEAD11, SlDEAD12, SlDEAD14, SlDEAD20, SlDEAD23, SlDEAD25, SlDEAD27, SlDEAD28, SlDEAD35, SlDEAD36, SlDEAD37, and SlDEAD42—making it the largest clade. The 42 SlDEAD proteins shared nine highly conserved motifs, namely, Q-motif, motif I, motif Ia, motif Ib, motif II, motif III, motif IV, motif V, and motif VI, which are arranged in a specific order (Figure 1, Supplementary Figure S1). Through a detailed examination of these motifs across the SlDEAD proteins (Supplementary Figure S1), it is evident that the majority of these motifs exhibit a high degree of conservation. Notably, with the exception of SlDEAD21 and SlDEAD39, where the first amino acid of the Q-motif is serine, the Q-motifs of the remaining 40 DEAD proteins commence with alanine. Single amino acid alteration also occurs in the first amino acid of motif Ia. In most of the 42 DEAD proteins, motif Ia has a sequence of SAT. However, the serine in this position is replaced by threonine in SlDEAD2, SlDEAD10, and SlDEAD39 and substituted by alanine in SlDEAD17. Moreover, in the remaining motifs, more than one amino acid substitution event can be observed.

**Figure 2:**
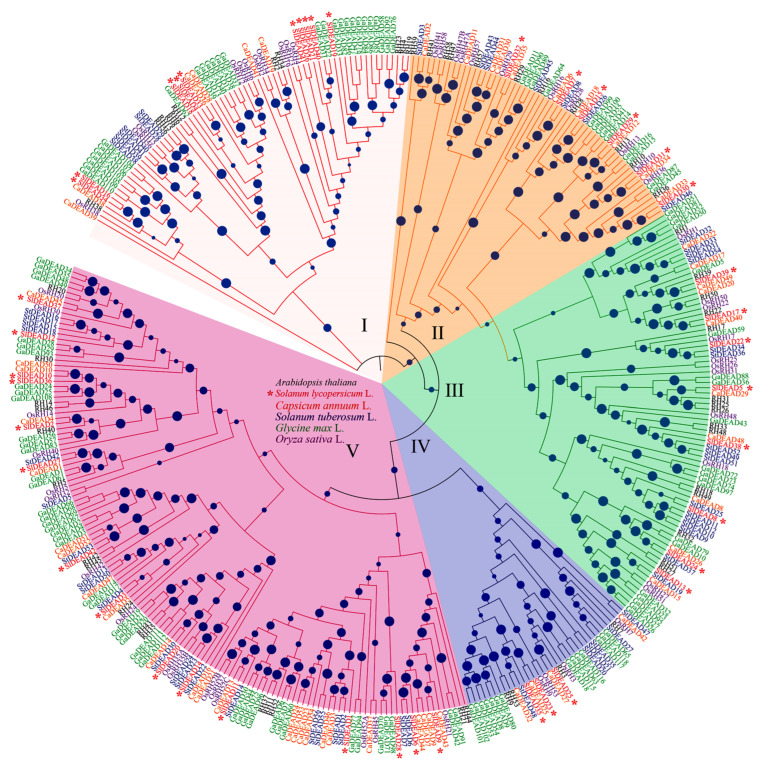
Phylogenetic tree of DEAD family genes derived from six species, including *Arabidopsis thaliana*, rice (*Oryza sativa*), soybean (Glycine max), and Solanaceae crops, like tomato (*Solanum lycopersicum*), pepper (*Capsicum annuum*), and potato (*Solanum tuberosum*). DEAD genes in tomato are indicated by *.

To investigate the phylogenetic relationships of DEAD families in tomato and other species, we additionally identified 57 *DEAD* genes from *Arabidopsis*, 118 from soybean (*Glycine max*), 53 from rice (*Oryza sativa*), 56 from potato (*Solanum tuberosum*), and 54 from pepper (*Capsicum annuum*) (Figure 2). Phylogenetic tree analysis based on the sequence alignment of these DEAD proteins showed that the 380 DEAD proteins were divided into five groups, namely, groups I to V (Figure 2). Among them, group V contains 136 DEAD proteins and thus forms the largest group. The SlDEAD proteins from clade IV (Figure 1) were further segregated into groups IV and V. Specifically, three of them, SlDEAD23, SlDEAD25, and SlDEAD35, were assigned to group IV, and the remaining 15 SlDEAD proteins were categorized into group V. SlDEAD proteins in the other clades shown in Figure 1 were correspondingly classified into groups I, II, and III when aligned with DEAD proteins from other species (Figure 2).

### 2.2. Collinearity Analysis and Chromosome Localization of SlDEAD Family Genes

**Figure 3:**
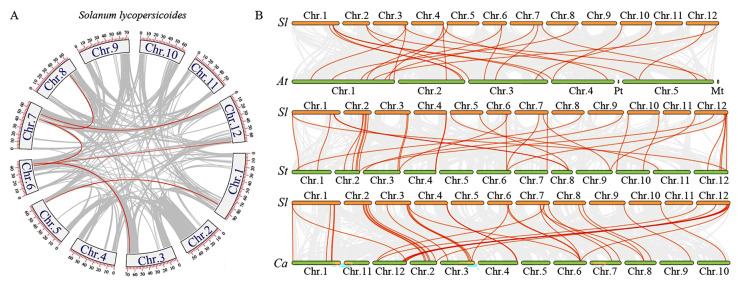
Collinearity analysis and chromosomal localization of *SlDEAD* genes in tomato. (**A**) Intra-species collinearity analysis of *SlDEAD* genes in tomato. The grey boxes represent the twelve chromosomes of tomato, and the numbers on the boxes represent the positions along the corresponding chromosomes. The gray lines denote the gene duplication pairs in the whole tomato genome, and the red lines highlight collinearity relationships of *SlDEAD* gene pairs. (**B**) Inter-species collinearity analysis of DEAD genes among tomato (Sl), *Arabidopsis* (At), *S. tuberosum* (St), and *C. annuum* (Ca). Grey lines represent the gene pair experiencing gene replication events between tomato and the other three species, and the red lines refer the gene pair experiencing gene replication events between the SlDEAD and DEAD genes from the other three species.

Genome-level duplication events contribute to the diversification of genes and gene families within the plant genome. Previous studies reveal that a recent large-scale duplication event in the *Solanaceae* family was shared by tomato and potato [44,45]. To explore the gene replication events of *SlDEAD* genes in tomato, an intra-species collinearity analysis of the *DEAD* gene family was performed (Figure 3A, Supplementary Table S2). The analysis identified six duplication events involving 11 genes. Specifically, *SlDEAD4* and *SlDEAD18*, *SlDEAD14* and *SlDEAD20*, *SlDEAD19* and *SlDEAD21*, *SlDEAD19* and *SlDEAD40*, *SlDEAD23* and *SlDEAD35*, and *SlDEAD21* and *SlDEAD26* occur in segment duplication events. Two tandem replication clusters were identified from the *SlDEAD* gene family, including *SlDEAD19* and *SlDEAD20* in chromosome 6 and *SlDEAD21* and *SlDEAD22* in chromosome 7. The collinearity between tomato and three other species, *Arabidopsis,* potato, and pepper, was also conducted to investigate the gene duplication events in the evolutionary history of *DEAD* family genes (Figure 3B and Supplementary Table S2). As shown in Figure 3B, a total of 22, 3, 7, and 32 paired collinearity relationships were found between 42 *SlDEAD* genes in tomato and 57 *AtDEAD* genes in *Arabidopsis*, 56 *StDEAD* genes in potato, and 54 *CaDEAD* genes in pepper. The result indicates that the *DEAD* gene family in tomato is more closely related to that of Solanaceae plants than to that of *Arabidopsis*.

**Figure 4:**
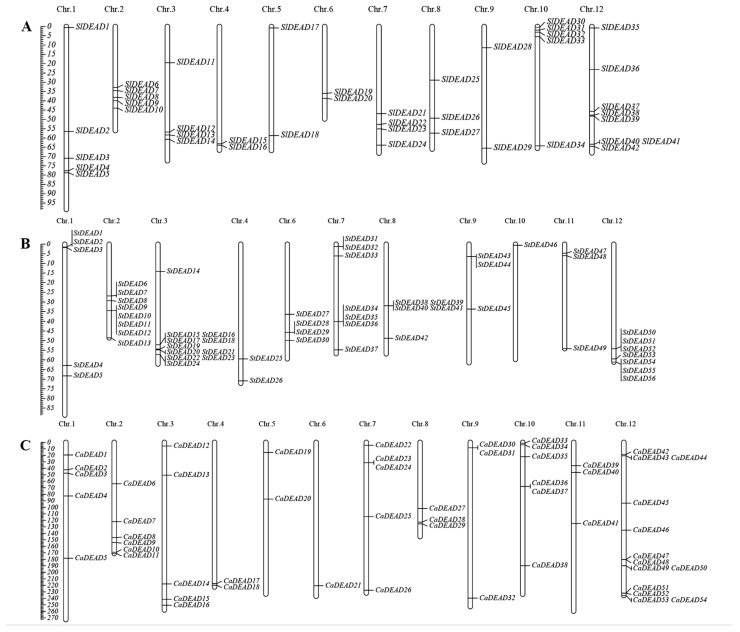
Distribution of the identified *DEAD* genes on twelve chromosomes of tomato (**A**), potato (**B**), and pepper (**C**). The chromosome number is denoted at the top of each chromosome, and gene names are presented on the right side of each chromosome. The scale on the left is calibrated in megabases (Mbs). Chr, chromosome.

According to the annotation information in the tomato genome, we found that the 42 *SlDEAD* genes were unevenly distributed across the 12 chromosomes (Figure 4). No correlation between the length of the chromosome and the number of *SlDEAD* genes was detected. The maximum number of eight *SlDEAD* genes was discovered on chromosome 12, followed by five *SlDEAD* genes on each of chromosomes 1, 2, and 10. However, no *SlDEAD* genes were mapped to chromosome 11. Multiple *SlDEAD* gene clusters were located at the distal end of the tomato chromosomes. For example, *SlDEAD30*, *SlDEAD31*, *SlDEAD32*, and *SlDEAD33* were clustered in the extreme end region of chromosome 10. A similar phenomenon was noted in potato and pepper. Clusters of *StDEAD* and *CaDEAD* genes were detected on diverse chromosomes within their genomes.

### 2.3. Gene Structure and Conserved Motif Analysis of Tomato SlDEAD Genes

**Figure 5:**
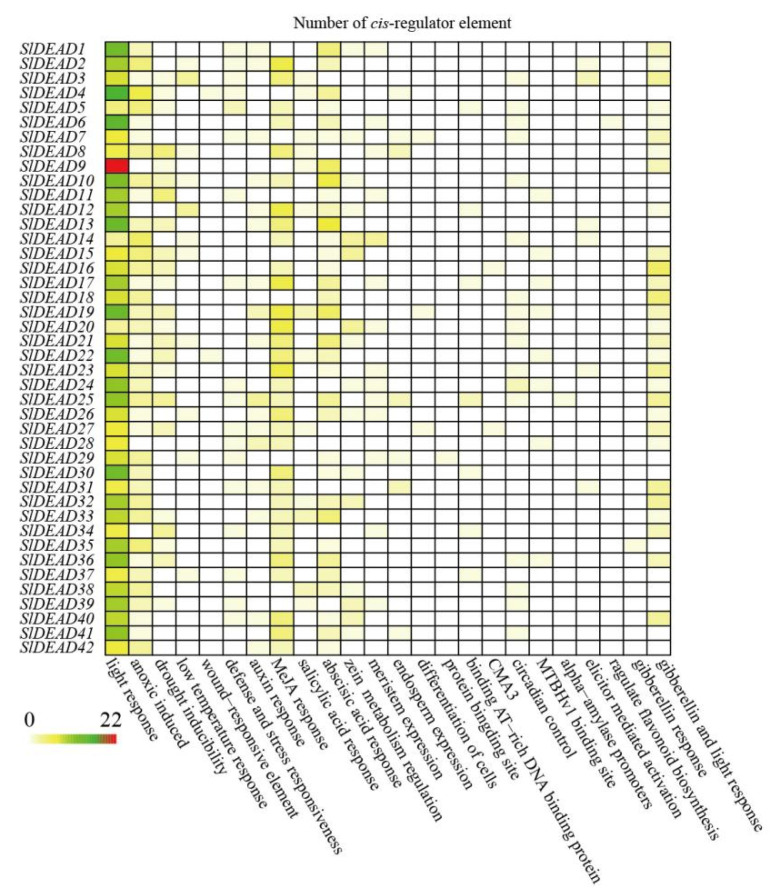
Cis-acting regulatory elements of the promoters of *SlDEAD* family genes. The analysis was conducted on the 2 kb upstream region of each genes using TBtools (v2.210) software. The distinct colors represent the quantity of each cis-acting element.

Among the 42 *SlDEAD* genes, only *SlDEAD6*, *SlDEAD9,* and *SlDEAD42* lack introns (Table 1). The remaining *SlDEAD* genes contain various numbers of introns. Specifically, *SlDEAD14* contains as many as 26 introns, while the others have at least one intron each (Table 1). The sequences of *SlDEAD18*, *SlDEAD28*, and *SlDEAD32* exceeded 10 kb in length, possibly due to the presence of relatively large introns. To explore the cis-regulatory elements responsible for their potential functions, the 2 kb DNA sequences upstream of the corresponding *SlDEAD* genes were analyzed (Figure 5). A large number of cis-acting elements, such as hormone response, light response, and stress-related elements, were identified in the promoter regions of *SlDEAD* genes. Among them, light response elements are the most prominent in all *SlDEAD* family genes, suggesting the expression of SlDEAD genes might be closely tied to light. Additionally, anoxic-induced elements and elements responsive to MeJA, ABA, and GA were detected in the majority of *SlDEAD* genes, suggesting a possible role of these SlDEAD proteins in hormone signaling and stress response.

### 2.4. Subcellular Localization of SlDEAD Proteins

**Figure 6:**
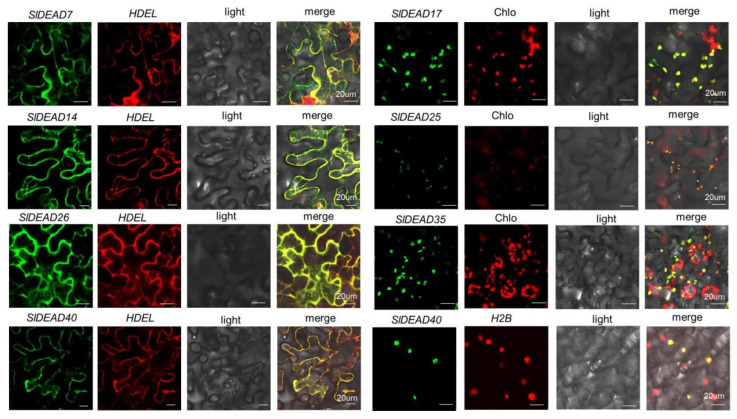
Subcellular localization of SlDEAD proteins. The SlDEAD proteins under investigation encompass SlDEAD7, SlDEAD14, SlDEAD17, SlDEAD25, SlDEAD26, SlDEAD35, and SlDEAD40. The endoplasmic reticulum is marked by HDEL-fused RFP, while the nucleus is labeled with RFP-fused H2B. The autofluorescence emanating from the chloroplast (Chlo) serves as an indicator for the presence and location of the chloroplast.

Subcellular localization prediction suggests that 27 SlDEAD proteins might be located in the nucleus, while the others are distributed in the cytoplasm, mitochondria, and chloroplast (Table 1). Since subcellular localization can provide additional clues into their potential functions, seven SlDEADs, including SlDEAD7, SlDEAD14, SlDEAD17, SlDEAD25, SlDEAD26, SlDEAD35, and SlDEAD40, were selected for experimental verification in conjunction with organelle marker genes (Figure 6). The results show that SlDEAD7, SlDEAD14, and SlDEAD26 were localized in the endoplasmic reticulum (ER), while SlDEAD17, SlDEAD25, and SlDEAD35 were targeted at the chloroplast. Only the localization of SlDEAD17 and SlDEAD25 was consistent with the localization prediction. On the other hand, SlDEAD40 was found to be located in both the ER and the nucleus. The results indicate that SlDEADs may perform diverse functions with specific localization in various organelles.

### 2.5. Expression Pattern of SlDEAD Genes in Response to High-Temperature Stress

**Figure 7:**
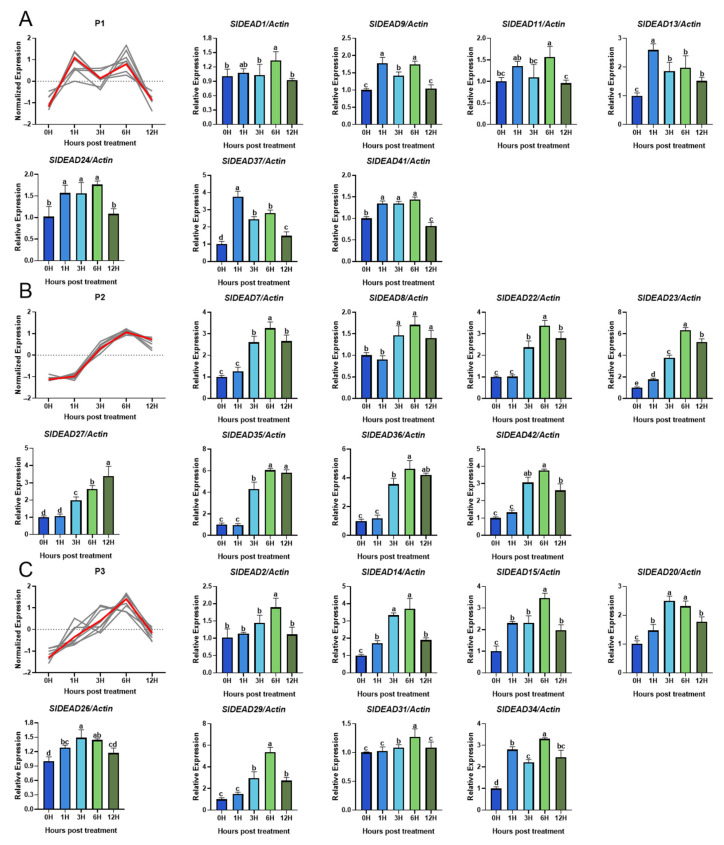
Expression profiles of *SlDEAD* family genes in tomato at 1, 3, 6, and 12 h post high-temperature stress treatment. (**A**–**C**) depict the expression pattern (the first panel) and relative expression levels (rest of the panels) of *SlDEAD* family genes in groups 1, 2, and 3. The y-axis in the first panel represents the normalized expression values derived from RT-qPCR results. The relative expression of each *SlDEAD* gene was calculated based on RT-qPCR data obtained from three independent biological replicates. Letters above the bars indicate significant differences (*p* < 0.05) calculated by Duncan’s new multiple range test.

**Figure 8:**
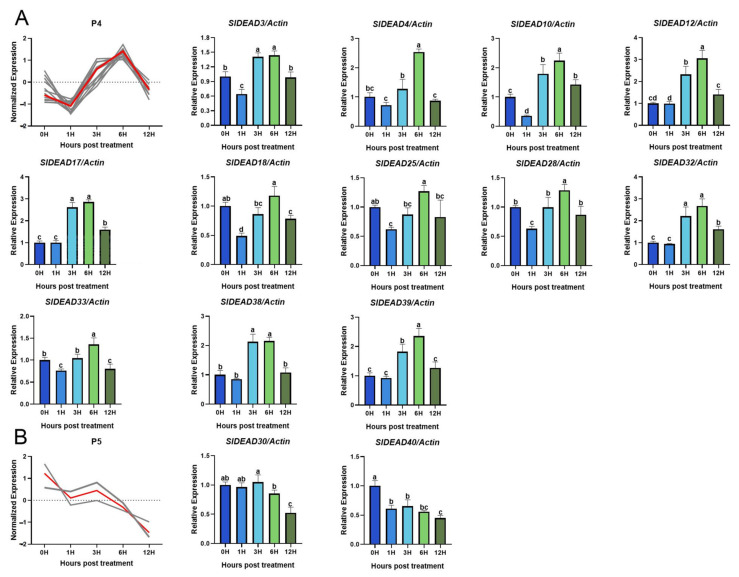
Expression profiles of *SlDEAD* family genes in tomato at 1, 3, 6, and 12 h post high-temperature stress treatment. (**A**,**B**) depict the expression pattern (the first panel) and relative expression levels (rest of the panels) of *SlDEAD* family genes in groups 4 and 5, respectively. The y-axis in the first panel represents the normalized expression values derived from RT-qPCR results. The relative expression of each *SlDEAD* gene was calculated based on RT-qPCR data obtained from three independent biological replicates. Letters above the bars indicate significant differences (*p* < 0.05) calculated by Duncan’s new multiple range test.

To determine whether these SlDEADs respond to heat stress, we analyzed their expression levels in tomato leaves during the early stage of high-temperature treatment. The expression levels of *SlDEAD5*, *SlDEAD6*, *SlDEAD12*, *SlDEAD16*, *SlDEAD19*, and *SlDEAD21* genes in tomato leaves were too low to be precisely detected under both normal and high-temperature stress conditions by RT-qPCR. These genes were thus excluded from the analysis. The RT-qPCR results (Figure 7 and Figure 8) reveal that the expression of all the remaining 37 *SlDEAD* genes responded to high-temperature treatment to varying extents. Based on their expression patterns at 1, 3, 6, and 12 h post-treatment (hpt) at high temperatures, we classified the 37 *SlDEAD* genes into five groups. Group 1 contained seven *SlDEAD* genes (Figure 7A), including *SlDEAD1*, *SlDEAD9*, *SlDEAD11*, *SlDEAD13*, *SlDEAD24*, *SlDEAD37*, and *SlDEAD41*. Their expression exhibited a dynamic pattern, with a significant increase at 1 or 6 hpt and a return to baseline expression at 12 hpt. Both groups 2 and 3 consisted of eight *SlDEAD* genes (Figure 7B,C). These *SlDEAD* genes were continuously activated during the first 12 h. The difference in the expression pattern between groups 2 and 3 was whether the expression of the genes at 12 hpt was significantly decreased compared to 6 hpt. Twelve *SlDEAD* genes, including *SlDEAD3*, *SlDEAD4*, *SlDEAD10*, *SlDEAD12*, *SlDEAD17*, *SlDEAD18*, *SlDEAD25*, *SlDEAD28*, *SlDEAD32*, *SlDEAD33*, *SlDEAD38*, and *SlDEAD39*, were classified into group 4. Their expression was suppressed at 1 hpt but then increased at 3 and 6 hpt. The other two *SlDEAD* genes, *SlDEAD30* and *SlDEAD40*, exhibited a continuous downregulation during the first 12 h. The results suggest that most of the SlDEAD family genes respond to heat stress, and the diverse expression patterns under heat stress may indicate their varied roles in the tomato’s response to heat stress.

## 3. Discussion

DEAD-box RNA helicases are a class of highly conserved enzymes that rely on their activities of ATPase to perform a broad range of functions in most RNA metabolic processes. Over the past few years, significant research efforts have been dedicated to understanding their functions and mechanisms. Notably, recent studies have reported that DEAD-box family proteins can modulate phase separation by regulating their own domains in an ATP-bound state, which, in turn, enables the selective recruitment or release of free proteins and nucleic acids within cells, as demonstrated in [46]. In this study, we identified 42 DEAD family genes from tomatoes and constructed evolutionary trees in comparison with 57 *DEAD* genes from *Arabidopsis*, 118 genes from soybean, 53 genes from rice, 56 genes from potato, and 54 genes from pepper. The presence of a large RNA helicase gene family across these species strongly suggests that RNA helicases are likely to play pivotal regulatory roles in multiple aspects of plant growth and development. From the results of phylogenetic and collinear analyses, it is evident that most DEAD genes within the same cluster share similar characteristics (Figure 1 and Figure 2). Specifically, 57 and 59 collinear pairs, respectively, were detected in the collinearity analysis between tomato and potato, as well as tomato and peppers. In contrast, a considerably lower number of collinear pairs was observed between tomato and *Arabidopsis*. The functional diversity of DEAD gene family members in tomato might be attributed to structural disparities among genes. For example, SlDEAD15 contains 18 introns, while SlDEDA6 and SlDEDA42 are intron-less. In the sequence comparison of SlDEAD, there were six pairs of segmental and tandem duplication events in tomatoes involving 11 related genes. Moreover, the number of cis-acting elements enriched in the promoter sequences of different genes exhibited substantial variation. Collectively, the tomato DEAD gene has maintained a certain level of conservation throughout the evolutionary history of plants, albeit accompanied by a degree of genetic variation, which likely contributes to the adaptive evolution and functional diversification of this gene family in the context of tomato plants.

The conserved motifs within the DEAD family genes play a crucial role in modulating gene function [28]. The DEAD family harbors nine conserved motifs that are arranged in a precise order. Among them, motifs II, I, Q, and VI are indispensable for ATP binding and hydrolysis. In contrast, motifs Ia, Ib, III, IV, and V exhibit lower specificity but may participate in RNA interaction and the remodeling of ribonucleic acid molecules [21]. Motif I is critical for ATPase and helicase activity. Mutations in either the first alanine, the conserved lysine, or the last threonine result in the loss of ATPase activity, primarily due to a reduction in the affinity and hydrolysis rate of motif I for ATP [47,48]. Based on the analysis of motifs in tomato SlDEADs, we found that the first amino acid in SlDEAD21 and SlDEAD39 is serine, whereas in other genes, it is alanine, with a high degree of conservation. This disparity might lead to alterations in helicase activity. Although motif Ia is not directly engaged in ATP binding and hydrolysis, it is essential for RNA binding in conjunction with motifs IV and V [49]. Among the tomato DEAD family proteins, the alanine within motif Ia is predominantly converted into cysteine (C), serine (S), and valine (V) (Supplementary Figure S1). As a pivotal motif, motif III is involved in the coupling of ATPase and helicase activities. The mutation of specific amino acids within this motif leads to a complete loss of helicase activity, while the effects on ATP binding, hydrolysis, and RNA binding are relatively minor [50]. It was reported that replacing serine and threonine of the Has1 motif III with alanine slightly reduced ATP binding, hydrolysis, and RNA binding but marginally diminished helicase activity [50]. The first amino acid of motif III in SlDEAD17 is alanine, whereas it is threonine in SlDEAD2, SlDEAD10, SlDEAD36, and SlDEAD39 (Supplementary Figure S1). Taken together, the conserved motifs in tomato SlDEADs deviate slightly from those of the known DEAD helicases. Whether these modifications directly impact binding to RNA needs further investigation.

In recent years, extensive investigations into DEAD-box RNA helicases have uncovered their multifunctional characteristics. Notably, these enzymes function as crucial mediators among diverse aspects of RNA metabolism and serve as central nodes connecting multiple cellular processes [28]. For instance, some DEAD-box proteins have been reported to participate in RNA processing and maturation and mRNA transport. The eukaryotic DEAD-box protein DDX17 binds to a specific subgroup of pre-miRNA containing the VCAUCH motif and promotes its maturation through the formation of a microprocessor complex [51]. The overexpression of *OsRH42* within the nucleus facilitates the splicing of pre-mRNA at low temperatures, thereby regulating the expression of stress-resistant genes under low-temperature conditions [33]. Except for their involvement in RNA processing, eIF4A-I and its closely related counterpart eIF4A-III, which are regarded as the smallest helicases, utilize their helicase activity to resolve the secondary structures in the 5′UTRs of mRNA during cap-dependent translation initiation [52]. eIF4A-III (DDX48) can be recruited by CTIF1 to the 5′ termini of mRNA bound by the nuclear-cap-binding complex (CBC), thereby enhancing translation [53].

Due to their roles in modulating RNA metabolism, DEAD-box RNA helicases are widely involved in plant responses to diverse stresses. A number of RNA helicases have been identified as highly promising candidates that endow plants with multiple stress tolerances. For example, OsABP in rice [54], PDH45 in chili peppers [55], and SlDEAD30 and SlDEAD31 [42] in tomatoes have the capacity to enhance tolerance to a variety of abiotic stresses such as drought, cold, and salinity. In this study, diverse cis-regulatory elements were identified within the SlDEAD genes (Figure 5). These results indicate that these genes may have distinct expression patterns under different conditions, encompassing growth, development, and stress-response aspects. These results imply that the SlDEAD family genes play a crucial role in regulating the tolerance of tomatoes to various stresses.

The subcellular localization of proteins is closely associated with their function. Distinct subcellular localizations define the specific physiological processes in which the corresponding proteins are involved. Previous research reveals that nucleus-localized OsRH42 helps in the splicing of pre-mRNA at low temperatures, thereby regulating cold-responsive gene expression [33]. The chloroplast-targeted DEAD-box RNA helicase SlDEAD39 participates in the processing of chloroplast 23S rRNA and affects the structure of cells and chloroplasts. Its mutant shows a chlorosis phenotype at the cotyledon stage with a reduced photosynthetic capacity and a significant inhibition of tomato growth [41]. Tomato SlRBP1 binds to a large number of RNAs related to chloroplast functions, channels RNA onto the SleIF4A2 translation initiation complex, and promotes the translation of its target RNAs to regulate chloroplast function. SlRBP1 knockdown mutants (*SlRBP1*) exhibit phenotypes of the reduced accumulation of total chlorophyll and impaired chloroplast ultrastructure [56]. Based on the result of subcellular localization prediction (Table 1), the majority of SlDEAD proteins were predominantly located in the nucleus, followed by chloroplasts, mitochondria, and cytoplasm. As shown in the subcellular localization utilizing transient expression in tobacco leaves, most of the tested SlDEAD proteins exhibited a different localization from the result of prediction. For example, SlDEAD40, which was predicted to be localized in the nucleus, was actually found in the endoplasmic reticulum in addition to the nucleus (Figure 6). It is thus proposed that SlDEAD40 might be involved in RNA splicing in the nucleus and RNA translation in the ER. Similarly, chloroplast-targeted SlDEAD35 may participate in the regulation of chloroplast RNA and affect plant photosynthesis under stress. Therefore, the results of subcellular localization provide a more precise basis for elucidating the functions of SlDEAD proteins.

The analysis of cis-acting elements that regulate gene expression patterns through interaction with various transcriptional factors and other regulatory molecules may reveal the potential to determine their abilities to regulate plants’ adaptation to various environmental stresses and developmental signals [57]. In this study, diverse cis-regulatory elements were identified within the *SlDEAD* genes (Figure 5), which indicates that these genes may have distinct expression patterns under different conditions, encompassing growth, development, and stress-response aspects. Notably, among the cis-acting elements within the SlDEAD genes, the quantity of light-responsive elements was the highest, suggesting that SlDEAD family genes may respond to light stimuli. Moreover, a number of stress-response elements, such as wound-responsive elements, elements related to drought inducibility, and anaerobic induction, as well as those associated with defense and stress responsiveness, were highly enriched. Additionally, several hormone response elements, including those responsible for auxin responsiveness, abscisic acid responsiveness, gibberellin-responsive elements, elements involved in zein metabolism regulation, and salicylic acid responsiveness, were abundantly present. These results imply that the SlDEAD family genes play a crucial role in regulating the tolerance of tomatoes to various stresses. Further verification of their expression patterns under various conditions is required in future studies.

High-temperature stress is one of the major abiotic stresses exacerbated by climate change. Under high-temperature stress, the gene expression within cells is significantly disrupted, leading to disorder in the overall transcription and translation processes of crops [58]. Heat stress can also trigger the formation of stress granules [59]. The formation and assembly of stress granules (SGs) are reversible, affording the cell the capacity to sequester mRNA in the form of complexes within SGs under adverse conditions. When normal conditions are restored, the mRNA can be released from SGs and may either enter the degradation pathway or re-enter the translation cycle [60]. Given their primary roles in regulating post-transcriptional processing and the stability of RNAs [31,61], DEAD-box RNA helicases are possibly a crucial factor in modulating plant tolerance to high-temperature stress. For example, high-temperature stress can suppress the expression of the DEAD-box RNA helicases *STRS1* and *STRS2* in *Arabidopsis* [62]. In the mutants of *STRS1* and *STRS2*, the transcriptional levels of the heat-responsive genes *HSP101*, *HSF4*, and *HSF7* were remarkably elevated, thereby enhancing the mutants’ tolerance to high temperatures. *eIF4E* is a highly characteristic RNA helicase in translation initiation [63]. After 30 min of heat stress, eIF4E and polyA^+^ mRNA become concentrated in cytoplasmic granules [64]. DEAD-box RNA helicases also act as RNA chaperones, actively resolving misfolded RNA structures. In rice, an increase in temperature directly augments helicase activity, and the expression of *TOGR1* (Thermo-Tolerant Growth Required1) helicase is closely related to diurnal temperature fluctuations [65]. The nucleus-localized TOGR1 was further found to interact with the small subunit (SSU) pre-rRNA processome, which facilitates the correct folding of pre-rRNA and thus maintains rRNA homeostasis at high temperatures [65].

The expression pattern of target genes under specific stress conditions can effectively assist in screening for relevant resistance genes. Building on this, genes that exhibit significant up- or downregulation in response to stress are likely to play crucial roles in the plants’ adaptive mechanisms. In this study, we investigated whether the expression of *SlDEAD* genes responds to high-temperature stress. Our investigation reveals that the expression levels of the majority of *SlDEAD* genes exhibited dynamic changes over time and presented diverse trends (Figure 7 and Figure 8). A subset of genes, exemplified by SlDEAD30 and SlDEAD40, was significantly downregulated under high-temperature induction. Their downregulation suggests they may have a negative impact on high-temperature tolerance. A similar phenomenon was also reported in DEAD genes in rice in response to various stresses, in which the expression level of OsRH53 in rice declined under abiotic stresses such as drought, salt, cold, UV, and ABA treatments [66]. The majority of SlDEAD family genes showed an increasing trend within the 12 h period of high-temperature treatment, although their response times were sequential. Additionally, some *SlDEAD* genes exhibited at least one expression peak, suggesting the expression of these *SlDEAD* genes was dynamically regulated in the early phase of heat stress. This may be in accordance with the function of DEAD family genes during continual changes in RNA metabolism and post-transcriptional modulation.

Temperature is one of the key physical parameters influencing life on the Earth. Over the past few decades, high-temperature stress has led to a 5.5% and 3.8% decline in global wheat and corn production, respectively [25]. For every 1 °C rise in the global average temperature, the global yields of wheat, rice, corn, and soybeans are expected to decrease by an average of 6.0%, 3.2%, 7.4%, and 3.1% [26]. Developing heat-tolerant varieties is thus one of the fundamental approaches for plants to cope with high-temperature stress. Through an extensive investigation of the early response of tomato DEAD-box RHs to heat stress, this study identifies a number of highly promising candidate genes for potential genetic engineering and marker-assisted breeding. Genetic engineering technologies such as CRISPR/Cas9 have revolutionized plant breeding by enabling the precise manipulation of target genes [67]. With the application of these advanced genetic tools, the identified candidate genes can be effectively integrated into the revolution, which will endow the genetically engineered tomatoes with enhanced heat tolerance.

## 4. Materials and Methods

### 4.1. Plant Materials and Heat Stress Treatment

Tomato seeds of Ailsa Craig were sown in pots and cultivated within a plant growth chamber under a photoperiod of 16 h of light (22000Lx) at 28 °C and 8 h of darkness at 18 °C. When the seedlings reached the three-leaf stage, the seedlings were transferred to a 40 °C incubator with a relative humidity of 70% for a 12 h treatment. Tomato leaves were harvested from three individual seedlings at 0, 1, 3, 6, 9, and 12 h after treatment. The harvested leaves were rapidly frozen in liquid nitrogen and then stored at −80 °C.

### 4.2. Analysis of DEAD Family Gene Structures

The phylogenetic tree of *DEAD* family genes identified in different species, including *Arabidopsis*, rice, soybean, tomato, pepper, and potato, was constructed. The sequence of each *DEAD* gene was downloaded from the NCBI online website (https://www.ncbi.nlm.nih.gov/ accessed on 30 March 2025) and then subjected to phylogenetic analysis using the MEGA7 maximum likelihood method. Interspecific and intraspecific gene collinearity analysis was carried out based on the genomic files downloaded from the BRAD database (http://brassicadb.cn/#/, accessed on 30 March 2025) and visualized using Circos. Maps of the tomato DEAD gene structure and chromosome localization were visualized using TBtools software (https://bio.tools/tbtools, accessed on 30 March 2025). The cis-element analysis within the promoter region of each *SlDEAD* gene was analyzed using PlantCARE (http://bioinformatics.psb.ugent.be/webtools/plantcare/html/, accessed on 30 March 2025) and visualized using TBtools software.

### 4.3. Analysis of SlDEAD Protein Properties

The protein sequence of each DEAD member was downloaded from the NCBI website. Their physicochemical properties, including their relative molecular weight, isoelectric point, and stability were analyzed using the online software on the website Expasy (https://www.expasy.org/ accessed on 13 October 2023). The conserved domain of each SlDEAD was analyzed by CD-Search (https://www.ncbi.nlm.nih.gov/Structure/bwrpsb/bwrpsb.cgi, accessed on 13 October 2023) and visualized using TBtools software. Identification of the conserved motifs in DEAD-box proteins [21,68] was performed using the online MEME Suite (https://meme-suite.org/meme/, accessed on 13 October 2023) [69].

### 4.4. RT-qPCR Analysis

Total RNA was isolated from leaves using the Trizol reagent according to the manufacturer’s instructions (Vazyme Biotech, Beijing, China; cat. No. 7E0661A4) and reverse transcribed to cDNA using a 5 x All-in-one qRT SuperMix (Vazyme Biotech, Beijing, China; cat. No. 7E782C3). RT-qPCR was performed using Power SYBR Green PCR Master Mix (Vazyme Biotech, Beijing, China; cat. No. Q312-02-AA) on a quantitative PCR system (qTower3, Analytik, Jena, Germany). The *Actin* gene was used as the internal control for the calculation of relative expression. The data were analyzed with the 2^−ΔΔCt^ method [70]. For each primer pair, three technical replicate RT-qPCR reactions were conducted. Detailed information on the primer sequences is presented in Supplementary Table S1.

### 4.5. Subcellular Localization Analysis

The full-length sequences of the *SlDEAD7*, *SlDEAD14*, *SlDEAD17, SlDEAD25*, *SlDEAD26*, *SlDEAD35*, and *SlDEAD40* genes were amplified using the specific primers shown in Supplementary Table S1 and cloned into pEarlyGate103 vector via gateway technology for GFP-fused expression. *Agrobacterium* GV3101 carrying GFP-DEAD fusion expression vectors were cultured overnight and adjusted to an OD_600_ of 0.8 for tobacco (*Nicotiana benthamiana*) leaf injection. The infected tobacco leaves were collected after 48 h and then observed under a laser confocal microscope (FluoView™ FV3000, Olympus, Tokyo, Japan) at a 40× objective magnification. Vectors with HDEL-RFP and H2B-RFP fusion proteins were used as the markers of the endoplasmic reticulum and nucleus, respectively [71]. The fluorescence images of GFPs and RFPs were captured under excitation laser wavelengths of 488 nm and 561 nm, respectively. To localize chloroplasts, the autofluorescence of the chloroplasts in the tobacco leaves was detected in the red wavelength range (600–720 nm).

### 4.6. Statistical Analysis

Statistical analysis was performed using SPSS Statistics (Version 17.0; SPSS Inc., Chicago, IL, USA). All data presented herein are the averages and standard errors derived from three biological replicates. The significant differences (*p*-value < 0.05) among samples were determined based on Duncan multiple range tests [72].

## 5. Conclusions

In this study, 42 *SlDEAD* genes were identified from the tomato genome, and a comprehensive analysis was conducted on their genetic relationships, chromosomal locations, conserved motifs, gene structures, and cis-regulatory elements, as well as their expression patterns. Despite the variations in the chromosome distribution, gene structure, and cis-elements among them, the 42 SlDEAD proteins all possess highly conserved domains. Taking the response to high-temperature stress as a case in point, it was found that, excluding the genes with low expression levels, the remaining 37 genes could be categorized into five groups based on the variation trends at different time points after high-temperature stress. After observing the subcellular localization of seven representative SlDEAD proteins, at least four of the SlDEAD genes were found to be located in the endoplasmic reticulum, three were located in the chloroplast, and one was located in the nucleus. These findings extend our understanding of the functional divergence and evolution of the DEAD gene family in tomato and reveal some candidate DEAD genes for future research on plant tolerance to abiotic stress.

## Figures and Tables

**Table 1:** Properties of tomato DEAD family members.

Gene Name	Gene ID	Genomic Position	Predicted Location	Amino Acid	Mass (Da)	Isoelectric Point	Coefficient of Instability	Number of Exons	Homologous Gene
SlDEAD1	solyc01g005960	626,619–632,574	cyto	604	65,709.62	8.16	41.9	6	AT2G42520.1
SlDEAD2	solyc01g057760	56,514,938–56,523,137	nucl	1221	135,061.40	9.96	63.63	11	AT3G06480.1
SlDEAD3	solyc01g079330	70,910,189–70,918,827	nucl	774	84,440.06	6.06	39.78	5	AT2G47330.1
SlDEAD4	solyc01g094350	77,623,834–77,631,727	nucl	498	56,905.28	8.73	45.84	9	AT4G00660.2
SlDEAD5	solyc01g095740	78,699,069–78,705,889	chlo	870	96,191.09	9.17	51.97	10	AT5G08610.1
SlDEAD6	solyc02g068190	32,785,826–32,787,814	nucl	662	75,669.38	9.27	40.07	1	AT2G33730.1
SlDEAD7	solyc02g070100	34,512,603–34,519,106	cyto	585	65,234.03	8.95	39.07	15	AT4G34910.1
SlDEAD8	solyc02g078880	38,098,218–38,103,721	nucl	594	66,304.59	9.04	41.17	11	AT5G05450.1
SlDEAD9	solyc02g081290	39,890,841–39,892,976	nucl	653	76,086.82	8.93	44.51	1	AT2G33730.1
SlDEAD10	solyc02g086660	43,885,348–43,891,471	nucl	706	77,150.90	9.33	52.1	8	AT2G33730.1
SlDEAD11	solyc03g052980	19,500,306–19,506,839	chlo	612	66,413.24	7.28	39.88	6	AT2G42520.1
SlDEAD12	solyc03g112350	56,827,775–56,836,207	nucl	651	70,294.39	9.85	46.22	10	AT5G63120.2
SlDEAD13	solyc03g114370	58,425,702–58,432,243	nucl	566	64,392.81	8.97	45.68	12	AT3G18600.1
SlDEAD14	solyc03g117440	60,661,466–60,664,137	cyto	595	66,647.42	6.11	45.02	27	AT5G51280.1
SlDEAD15	solyc04g081580	63,117,504–63,123,510	nucl	744	84,016.41	8.35	45.75	19	AT4G16630.1
SlDEAD16	solyc04g082790	63,926,584–63,931,290	nucl	499	55,268.09	5.26	36.21	8	AT3G53110.1 (LOS4)
SlDEAD17	solyc05g006130	839,121–843,666	chlo	559	62,335.58	6.5	46.64	7	AT1G59990.1
SlDEAD18	solyc05g048850	58,709,311–58,724,460	nucl	537	61,075.08	8.76	38.23	10	AT4G00660.2
SlDEAD19	solyc06g062800	36,012,777–36,014,915	nucl	413	46,827.74	5.58	43.31	4	AT1G54270.1
SlDEAD20	solyc06g068280	38,699,501–38,702,707	cyto	595	66,829.38	6.09	48.06	3	AT5G51280.1
SlDEAD21	solyc07g040750	46,856,884–46,859,084	nucl	413	46,659.80	5.21	47.44	4	AT1G54270.1
SlDEAD22	solyc07g042270	52,721,025–52,727,332	nucl	597	67,629.27	9.54	38.7	10	AT2G40700.1
SlDEAD23	solyc07g044760	55,132,119–55,137,026	chlo	630	67,146.29	9.43	33.14	7	AT3G22330.1
SlDEAD24	solyc07g064520	63,853,690–63,858,103	nucl	754	85,281.23	8.97	42.64	8	AT5G54910.1
SlDEAD25	solyc08g042050	28924,652–28934,324	chlo	746	81,518.47	6.26	46.94	10	AT5G26742.2
SlDEAD26	solyc08g062800	49,300,134–49,301,991	nucl	413	46,887.76	5.54	48.39	4	AT1G54270.1
SlDEAD27	solyc08g076200	57,382,754–57,390,648	nucl	553	61,228.04	8.75	33.96	13	AT1G31970.1 (STRS1)
SlDEAD28	solyc09g015930	11,382,872–11,400,498	chlo	558	60,842.18	6.62	46.01	14	AT3G58570.1
SlDEAD29	solyc09g090740	65,522,411–65,531,624	nucl	799	90,445.84	9.06	51.31	15	AT3G16840.1
SlDEAD30	solyc10g005520	426,164–431,795	nucl	488	54,692.30	8.53	38.21	6	AT1G16280.1
SlDEAD31	solyc10g007550	1,854,816–1,862,972	nucl	439	48,897.70	9.16	44.83	13	AT5G60990.1
SlDEAD32	solyc10g009070	3,093,425–3,105,024	cyto	785	87,843.93	9.9	36.06	11	AT1G77030.1
SlDEAD33	solyc10g017530	5,410,295–5,419,584	chlo	791	55,720.00	8.66	40.29	9	AT4G00660.2
SlDEAD34	solyc10g085790	64,194,367–64,199,371	nucl	508	56,255.08	5.22	36.31	8	AT3G53110.1 (LOS4)
SlDEAD35	solyc12g006320	843,041–848,893	mito	643	69,762.97	9.56	43.14	7	AT3G22330.1
SlDEAD36	solyc12g035130	23,234,550–23,240,564	nucl	612	66,663.87	9.7	47.16	8	AT3G01540.2
SlDEAD37	solyc12g044860	45,651,195–45,658,063	nucl	479	53,288.40	8.75	48.47	10	AT1G55150.1
SlDEAD38	solyc12g056340	47,655,899–47,661,775	chlo	805	92,434.54	8.89	48.82	10	AT1G63250.1
SlDEAD39	solyc12g056740	48,175,584–48,181,629	chlo	636	70,077.55	9.88	49.59	9	AT4G09730.1
SlDEAD40	solyc12g095990	63,417,531–63,419,666	nucl	413	46,843.66	5.46	47.67	4	AT1G54270.1
SlDEAD41	solyc12g096000	63,425,877–63,428,469	nucl	394	44,850.52	5.64	46.25	4	AT1G54270.1
SlDEAD42	solyc12g098700	64,461,108–64,464,551	nucl	1147	130,890.60	6	38.69	1	AT1G20920.1

## Data Availability

The original contributions presented in this study are included in the article and Supplementary Materials.

## Supplementary Materials

The following supporting information can be downloaded at: https://www.mdpi.com/article/10.3390/plants14081172/s1, Table S1: List of primer sequences used in the study. Table S2: List of gene duplication pairs of *SlDEAD* genes between tomato and other species. Figure S1: Sequence alignment of 42 SlDEAD proteins identified from tomato genome.
