# Supplementary material for: Characterization and Early Response of the DEAD Gene Family to Heat Stress in Tomato
Source: Plants (Basel). 2025 Apr 9;14(8):1172. doi: 10.3390/plants14081172 (PMC12030476; doi:10.3390/plants14081172)
Supplement: Supplementary file 1 [file plants-14-01172-s001.zip › Supplementary Figure S1.pdf]

Supplemental Figure S1

Sequence alignment of 42 DEAD proteins identified from tomato genome. The locations of Q-motif, motif I, motif Ia, motif Ib, motif II, motif III, motif IV, motif V, and motif VI are indicated in colored boxes.

```
                                                                    motif IV

SlDEAD17—101264928    VVETNTNETFFAEESVSWT—SLGVSESLSRALSS-SGLHRPSLIQAACIPSI-LSG—V   102
SlDEAD7—101255555     ——EGRVEIEEDEEQTFE—ELGLDPRLIRALTK-KTIDKPTPIQRVAIPLI-LEG—K   53
SlDEAD39—101257152    ——GGVKKSKKKAVASSFE—ELGLTEEVMGALGE-MGISEPTEIQSIGIPAV-IEG—K   149
SlDEAD29—101265585    ———EQDSVDETEYYAWN—ELRLHPLLMKSIYA-LKFKEPTPIQQACIPAGSHQG—K   208
SlDEAD8—101259209     ——MASANQNKALTNTRFSDLEPRLAEPVLEALTN-SGFEFCTPVQAATIPLL-CSY—K   53
SlDEAD32—101246967    AELKRREKQKKKAKSGGFE—SLGLSSNIFRGIKR-KGYRVPTPIQRKTMPLI-LSG—F   62
SlDEAD15—101266117    F——FASSEGVTFHANSFI—ELHISRPLLRACEA-LGYSKPTPIQAACIPLA-LTG—R   178
SlDEAD30—101259679    KQLDKETNSGPTPSYITFS—DLGLAGWAVQTCNE-LGMKKPTPVQYHCIPRI-LSG—Q   90
SlDEAD31—101257016    ————MDESKEETKSFK—ELGVCDQLIEACDN-LGWKNPSKIQAEAMPHA-FEG—K   47
SlDEAD14—101259928    —DGED——IPPPIKTFK—EMRFPEPMLKKLKA-KGIIQPTPIQVQGLPVI-LSG—R   188
SlDEAD20—101260827    —EGDD——VTPPIKNFK—DMRFPEPILKKLRA-KGIIQPTPIQVQGLPVI-LSG—R   188
SlDEAD6—101245423     —KGSR——IPRPMRNWA—ESALTTELLKAVER-AGYRKPSPIQMASIPLG-LQL—R   280
SlDEAD9—101267079     —KGSR——IPRPMRNWV—ESKLTTELLKAVDR-AGYKKPSPIQMAAIPLG-LQQ—R   329
SlDEAD1—101244218     —SGDN——VPPPVNTFA—EIDLGEAVNENIRR-CKYVKPTPVQRHAIPIS-LAG—R   182
SlDEAD11—101263192    —SGEN——VPPPVNTFA—EIDLGEAVNLNIRR-CKYVKPTPVQRYAIPIS-LAG—R   191
SlDEAD28—101258585    —NVSPVSCPAPSPIESFP—DMCLHASIMKDIEK-HGYTAPTSIQAQAMPVA-LSG—R   152
SlDEAD27—101253217    —TGKGANELKYKALEKFV—DAGLPSEVLECC——KNFEKPSPIQSHSWPFL-LDG—R   172
SlDEAD2—101266555     —TGGD——VPAPFMTFE—DTGFPPEILREIQF-AGFTSPTPIQAQTWPIA-LQN—R   612
SlDEAD10—01254752     —SGGD——VPPPFTSFE—ATGFPSEILREIHQ-AGFPAPSPIQAQSWPIA-LQG—R   191
SlDEAD36—101248651    —TGGD——VPAPLTSFE—ATGFPSEIVREMRQ-AGFSAPTPIQAQSWPVA-LQG—R   184
SlDEAD42—101254580    —HGKD——VPKPIKTWH—QTGLSSKMLDTIKK-LNYEKPMSIQAQALPVI-MSG—R   539
SlDEAD3—101264757     —SGFD——VPRPIKTFE—DTGFSAELMKAISK-QGYERPTPIQCQALPIV-LSG—R   267
SlDEAD12—101265421    —EGQD——VPRPIQMFH—EANFPDYCLEVISR-LGFVEPTPIQSQGWPMA-LKG—R   268
SlDEAD37—544216       —EGRD——VPKPIKSFH—DVGFPDYVLQEIEK-AGFTEPTPIQAQGWPMA-LKG—R   134
SlDEAD16—101243705    —SGDT——MYKSAKRFE—DLNLSPELLKGLYVQMQFERPSKIQEISLPMI-LTPPYK   132
SlDEAD34—101252541    —SGDT——MYKSAKRFE—DLNLSPELLKGLYVQMQFERPSKIQEISLPMI-LTPPYK   140
SlDEAD25—101263737    —EDNE——SNEDELSVS—KLGLPHRLVDALEK-RGITQLFPIQRAVLVPA-LEG—R   143
SlDEAD23—101249660    —N-LP——EDDEGLEIS—KLGISQEIVSALEK-KGITKLFPIQRAVLEPA-MQG—S   135
SlDEAD35—101259710    —ELEE——DCEEGLEIS—KLGISEEIVSALAQ-RGITSLFPIQRAVLEPA-MQG—S   149
SlDEAD21—104648456    —S————YDEAYDTFD—AMELKEDLLKGIYA-YGFEKPSAIQQRGIVPF-CKG—L   78
SlDEAD19—101244494    —S————YDEVYDSFD—AMGLQENLLRGIYA-YGFEKPSAIQQRGIVPF-CKG—L   78
SlDEAD40—101266108    —S————YDEVYDSFD—AMGLQENLLRGIYA-YGFEKPSAIQQRGIVPF-CKG—L   78
SlDEAD26—101252510    —S————YDEVHDSFD—AMGLQENLLRGIYA-YGFEKPSAIQQRGIVPF-CKG—L   78
SlDEAD41—101266405    —S————YDEVHDSFD—AMGLQENLLRGIYA-YGFEKPSAIQQRGIVPF-CKG—L   59
SlDEAD18—101258495    —SKDV——TATKGNDFE—DYFLKRELLMGIYE-KGFERPSPIQEESIPIV-LTG—S   201
SlDEAD4—101253227     —TEDV——TATKGNEFE—DYFLKRELLMGIYE-KGFERPSPIQEESIPIA-LTG—S   162
SlDEAD33—101251630    —TEDV——TATKGNEFE—DYFLKRELLMGIYE-KGFERPSPIQEESIPIA-LTG—S   155
SlDEAD22—101245966    —GNSE——VFASC-TFE—SLGLHTTLCDQLKERLGFEAPTLVQAQAIPVI-LSG—R   66
SlDEAD5—101245996     —GKSD——SHLTETRFD—QFPLS-PLSLKGVKDAGYKTMTVVQEATLPII-LKG—K   440
SlDEAD38—101259722    —DEKE——TILSSKRFD—EYDVS-PLTVKALTAAGYVQMTKVQEATLSTC-LEG—K   375
SlDEAD13—101245343    —KSGS——GIMSSELFS—SVQIS-ELTRKAIEDMGFEYMTQIQARAIPPL-VEG—K   117
SlDEAD24—101249838    —GSFS——RYAGCDRFS—QLPVS-KKTKDGLTQCKYKTMTDIQRASLPHS-LCG—R   106
                                       .                  :*
```

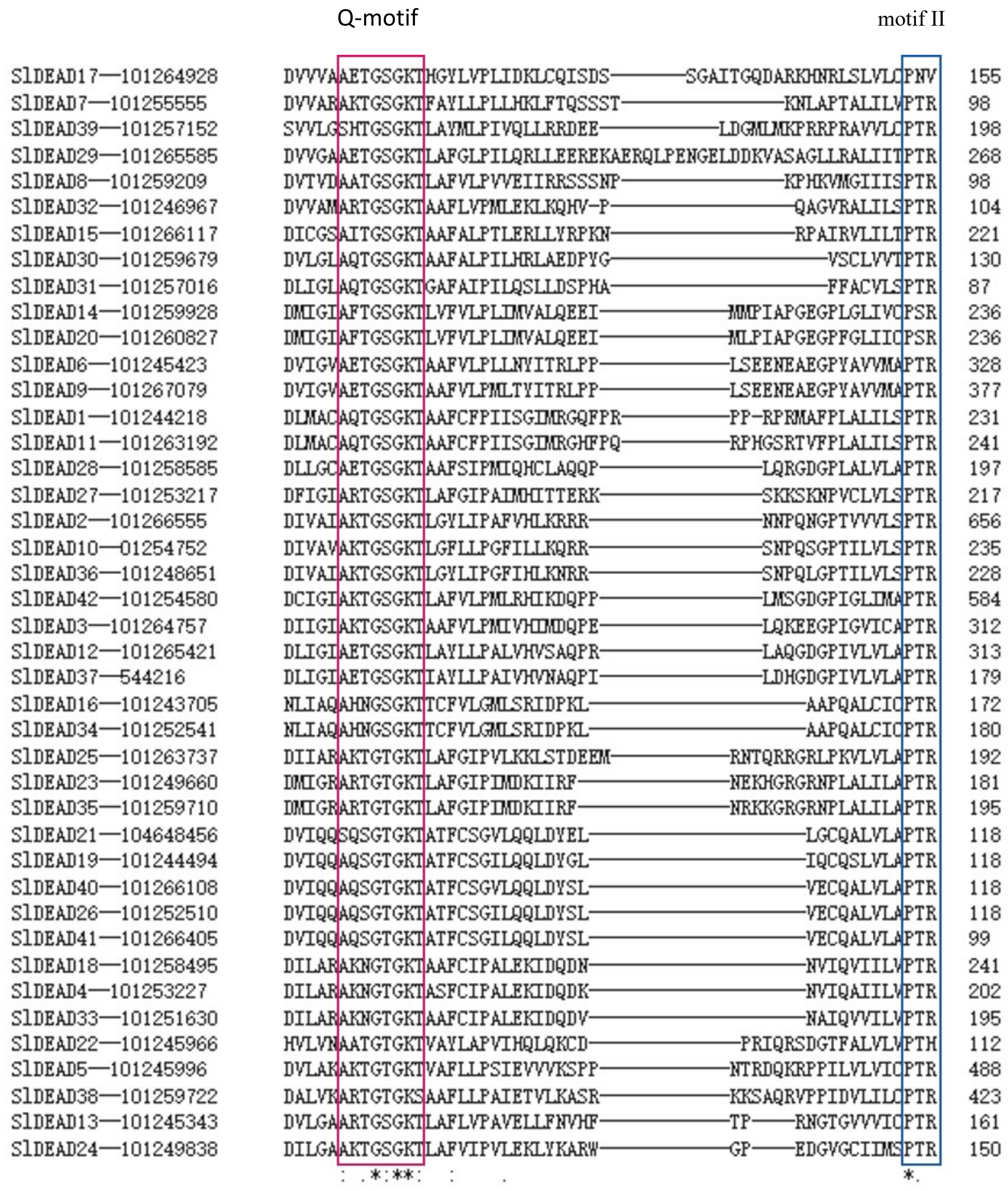
Q-motif
motif II
SlDEAD17—101264928 DVVVAAETGSGKTHGYLVPLIDKLCQISDS—SGAITGQDARKHNRLSLVLCPNV 155
SlDEAD7—101255555 DVVARAKTGSGKTFAYLLPLLHKLFTQSSST—KNLAPTALILVPTR 98
SlDEAD39—101257152 SVVLGSHTGSGKTLAYMLPIVQLLRRDEE—LDGMLMKPRRPRAVVLCPTR 198
SlDEAD29—101265585 DVVGAAETGSGKTLAFGLPILQRLLEEREKAERQLPENGELDDKVASAGLLRALIITPTR 268
SlDEAD8—101259209 DVTVDAATGSGKTLAFVLPVVEIIRRSSSNP—KPHKVMGIIISPTR 98
SlDEAD32—101246967 DVVAMARTGSGKTAAFLVPMLEKLKQHV-P—QAGVRALILSPTR 104
SlDEAD15—101266117 DICGSAITGSGKTAAFALPTLERLLYRPKN—RPAIRVLILTPTR 221
SlDEAD30—101259679 DVLGLAQTGSGKTAAFALPILHRLAEDPYG—VSCLVVTPTR 130
SlDEAD31—101257016 DLIGLAQTGSGKTGAFAIPILQSLLDSPHA—FFACVLSPTR 87
SlDEAD14—101259928 DMIGIAFTGSGKTLVFVLPLIMVALQEEI—MMPIAPGEGPLGLIVCPSR 236
SlDEAD20—101260827 DMIGIAFTGSGKTLVFVLPLIMVALQEEI—MLPIAPGEGPFGLIICPSR 236
SlDEAD6—101245423 DVIGVAETGSGKTAAFVLPLLNYITRLPP—LSEENEAEGPYAVVMAPTR 328
SlDEAD9—101267079 DVIGVAETGSGKTAAFVLPMLTYITRLPP—LSEENEAEGPYAVVMAPTR 377
SlDEAD1—101244218 DLMACAQTGSGKTAAFCFPIISGIMRGQFPR—PP-RPRMAFPLALILSPTR 231
SlDEAD11—101263192 DLMACAQTGSGKTAAFCFPIISGIMRGHFPQ—RPHGSRTVFPLALILSPTR 241
SlDEAD28—101258585 DLLGCAETGSGKTAAFSIPMIQHCLAQQP—LQRGDGPLALVLAPTR 197
SlDEAD27—101253217 DFIGIARTGSGKTLAFGIPAIMHITTERK—SKKSKNPVCLVLSPTR 217
SlDEAD2—101266555 DIVAIAKTGSGKTLGYLIPAFVHLKRRR—NNPQNGPTVVVLSPTR 656
SlDEAD10—01254752 DIVAVAKTGSGKTLGFLLPGFILLKQRR—SNPQSGPTILVLSPTR 235
SlDEAD36—101248651 DIVAIAKTGSGKTLGYLIPGFIHLKNRR—SNPQLGPTILVLSPTR 228
SlDEAD42—101254580 DCIGIAKTGSGKTLAFVLPMLRHIKDQPP—LMSGDGPIGLIMAPTR 584
SlDEAD3—101264757 DIIGIAKTGSGKTAAFVLPMIVHIMDQPE—LQKEEGPIGVICAPTR 312
SlDEAD12—101265421 DLIGIAETGSGKTLAYLLPALVHVSAQPR—LAQGDGPIVLVLAPTR 313
SlDEAD37—544216 DLIGIAETGSGKTIAYLLPAIVHVNAQPI—LDHGDGPIVLVLAPTR 179
SlDEAD16—101243705 NLIAQAHNGSGKTTCFVLGMLSRIDPKL—AAPQALCICPTR 172
SlDEAD34—101252541 NLIAQAHNGSGKTTCFVLGMLSRIDPKL—AAPQALCICPTR 180
SlDEAD25—101263737 DIIARAKTGTGKTLAFGIPVLKKLSTDEEM—RNTQRRGRLPKVLVLAPTR 192
SlDEAD23—101249660 DMIGRARTGTGKTLAFGIPIMDKIIRF—NEKHGRGRNPLALILAPTR 181
SlDEAD35—101259710 DMIGRARTGTGKTLAFGIPIMDKIIRF—NRKKGRGRNPLALILAPTR 195
SlDEAD21—104648456 DVIQQSQSGTGKTATFCSGVLQQLDYEL—LGCQALVLAPTR 118
SlDEAD19—101244494 DVIQQAQSGTGKTATFCSGILQQLDYGL—IQCQSLVLAPTR 118
SlDEAD40—101266108 DVIQQAQSGTGKTATFCSGVLQQLDYSL—VECQALVLAPTR 118
SlDEAD26—101252510 DVIQQAQSGTGKTATFCSGILQQLDYSL—VECQALVLAPTR 118
SlDEAD41—101266405 DVIQQAQSGTGKTATFCSGILQQLDYSL—VECQALVLAPTR 99
SlDEAD18—101258495 DILARAKNGTGKTAAFCIPALEKIDQDN—NVIQVIILVPTR 241
SlDEAD4—101253227 DILARAKNGTGKTASFCIPALEKIDQDK—NVIQAIILVPTR 202
SlDEAD33—101251630 DILARAKNGTGKTAAFCIPALEKIDQDV—NAIQVVILVPTR 195
SlDEAD22—101245966 HVLVNAATGTGKTVAYLAPVIHQLQKCD—PRIQRSDGTFALVLVPTH 112
SlDEAD5—101245996 DVLAKAKTGTGKTVAFLLPSIEVVVKSPP—NTRDQKRPPILVLVICPTR 488
SlDEAD38—101259722 DALVKARTGTGKSAAFLLPAIETVLKASR—KKSAQRVPPIDVLILCPTR 423
SlDEAD13—101245343 DVLGAARTGSGKTLAFLVPAVELLFNVHF—TP—RNGTGVVVICPTR 161
SlDEAD24—101249838 DILGAAKTGSGKTLAFVIPVLEKLYKARW—GP—EDGVGCIIMSPTR 150

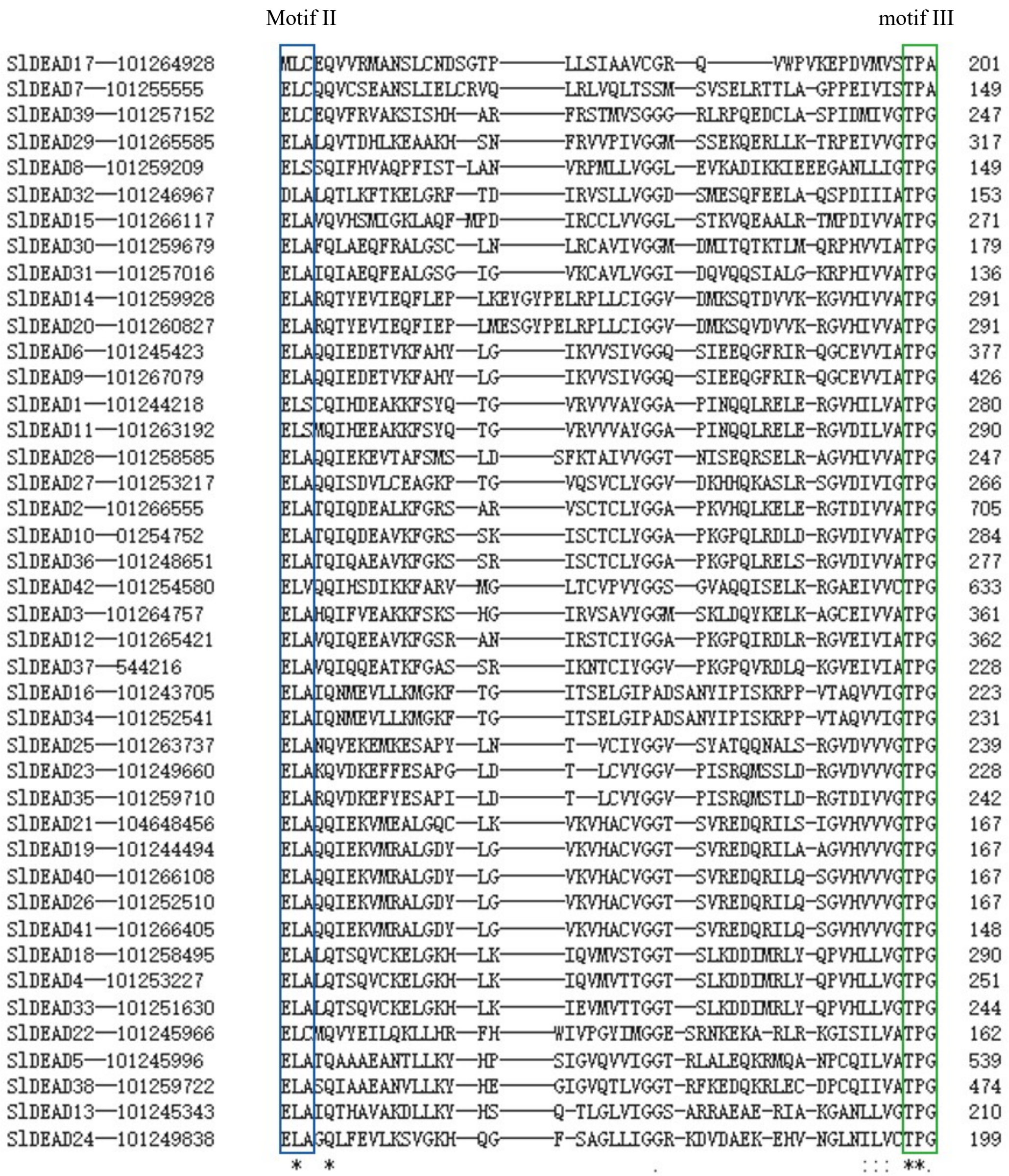

Motif II
motif III
SlDEAD17—101264928 MLCEQVVRMANSLCNDSGTP--------LLSIAAVCGR--Q--------VWPVKEPDVMVSTPA 201
SlDEAD7—101255555 ELCQQVCSEANSLIELCRVQ--------LRLVQLTSSM--SVSELRTTLA-GPPEIVISTPA 149
SlDEAD39—101257152 ELCEQVFRVAKSISHH--AR--------FRSTMVSGGG--RLRPQEDCLA-SPIDMIVGTPG 247
SlDEAD29—101265585 ELALQVTDHLKEAAKH--SN--------FRVVPIVGGM--SSEKQERLLK-TRPEIVVGTPG 317
SlDEAD8—101259209 ELSSQIFHVAQPFIST-LAN--------VRPMLLVGGL--EVKADIKKIEEEGANLLIGTPG 149
SlDEAD32—101246967 DLALQTLKFTKELGRF--TD--------IRVSLLVGGD--SMESQFEELA-QSPDIIIATPG 153
SlDEAD15—101266117 ELAVQVHSMIGKLAQF-MPD--------IRCCLVVGGL--STKVQEAALR-TMPDIVVATPG 271
SlDEAD30—101259679 ELAFQLAEQFRALGSC--LN--------LRCAVIVGGM--DMITQTKTLM-QRPHVVIATPG 179
SlDEAD31—101257016 ELAIQIAEQFEALGSG--IG--------VKCAVLVGGI--DQVQQSIALG-KRPHIVVATPG 136
SlDEAD14—101259928 ELARQTYEVIEQFLEP--LKEYGYPELRPLLCIGGV--DMKSQTDVVK-KGVHIVVATPG 291
SlDEAD20—101260827 ELARQTYEVIEQFIEP--LMESGYPELRPLLCIGGV--DMKSQVDVVK-RGVHIVVATPG 291
SlDEAD6—101245423 ELAQQIEDETVKFAHY--LG--------IKVVSIVGGQ--SIEEQGFRIR-QGCEVVIATPG 377
SlDEAD9—101267079 ELAQQIEDETVKFAHY--LG--------IKVVSIVGGQ--SIEEQGFRIR-QGCEVVIATPG 426
SlDEAD1—101244218 ELSCQIHDEAKKFSYQ--TG--------VRVVVAYGGA--PINQQLRELE-RGVHILVATPG 280
SlDEAD11—101263192 ELSMQIHEEAKKFSYQ--TG--------VRVVVAYGGA--PINQQLRELE-RGVDILVATPG 290
SlDEAD28—101258585 ELAQQIEKEVTAFSMS--LD-------SFKTAIVVGGT--NISEQRSELR-AGVHIVVATPG 247
SlDEAD27—101253217 ELAQQISDVLCEAGKP--TG--------VQSVCLYGGV--DKHHQKASLR-SGVDIVIGTPG 266
SlDEAD2—101266555 ELATQIQDEALKFGRS--AR--------VSCTCLYGGA--PKVHQLKELE-RGTDIVVATPG 705
SlDEAD10—01254752 ELATQIQDEAVKFGRS--SK--------ISCTCLYGGA--PKGPQLRDLD-RGVDIVVATPG 284
SlDEAD36—101248651 ELATQIQAEAVKFGKS--SR--------ISCTCLYGGA--PKGPQLRELS-RGVDIVVATPG 277
SlDEAD42—101254580 ELVQQIHSDIKKFARV--MG--------LTCVPVYGGS--GVAQQISELK-RGAEIVVCTPG 633
SlDEAD3—101264757 ELAHQIFVEAKKFSKS--HG--------IRVSAVYGGM--SKLDQYKELK-AGCEIVVATPG 361
SlDEAD12—101265421 ELAVQIQEEAVKFGSR--AN--------IRSTCIYGGA--PKGPQIRDLR-RGVEIVIATPG 362
SlDEAD37—544216 ELAVQIQQEATKFGAS--SR--------IKNTCIYGGV--PKGPQVRDLQ-KGVEIVIATPG 228
SlDEAD16—101243705 ELAIQNMEVLLKMGKF--TG--------ITSELGIPADSANYIPISKRPP-VTAQVVIGTPG 223
SlDEAD34—101252541 ELAIQNMEVLLKMGKF--TG--------ITSELGIPADSANYIPISKRPP-VTAQVVIGTPG 231
SlDEAD25—101263737 ELANQVEKEMKESAPY--LN--------T--VCIYGGV--SYATQQNALS-RGVDVVVGTPG 239
SlDEAD23—101249660 ELAKQVDKEFFESAPG--LD--------T--LCVYGGV--PISRQMSSLD-RGVDVVVGTPG 228
SlDEAD35—101259710 ELARQVDKEFYESAPI--LD--------T--LCVYGGV--PISRQMSTLD-RGTDIVVGTPG 242
SlDEAD21—104648456 ELAQQIEKVMEALGQC--LK--------VKVHACVGGT--SVREDQRILS-IGVHVVVGTPG 167
SlDEAD19—101244494 ELAQQIEKVMRALGDY--LG--------VKVHACVGGT--SVREDQRILA-AGVHVVVGTPG 167
SlDEAD40—101266108 ELAQQIEKVMRALGDY--LG--------VKVHACVGGT--SVREDQRILQ-SGVHVVVGTPG 167
SlDEAD26—101252510 ELAQQIEKVMRALGDY--LG--------VKVHACVGGT--SVREDQRILQ-SGVHVVVGTPG 167
SlDEAD41—101266405 ELAQQIEKVMRALGDY--LG--------VKVHACVGGT--SVREDQRILQ-SGVHVVVGTPG 148
SlDEAD18—101258495 ELALQTSQVCKELGKH--LK--------IQVMVSTGGT--SLKDDIMRLY-QPVHLLVGTPG 290
SlDEAD4—101253227 ELALQTSQVCKELGKH--LK--------IQVMVTTGGT--SLKDDIMRLY-QPVHLLVGTPG 251
SlDEAD33—101251630 ELALQTSQVCKELGKH--LK--------IEVMVTTGGT--SLKDDIMRLY-QPVHLLVGTPG 244
SlDEAD22—101245966 ELCMQVYEILQKLLHR--FH-------WIVPGYIMGGE-SRNKEKA-RLR-KGISILVATPG 162
SlDEAD5—101245996 ELATQAAAEANTLLKY--HP-------SIGVQVVIGGT-RLALEQKRMQA-NPCQILVATPG 539
SlDEAD38—101259722 ELASQIAAEANVLLKY--HE-------GIGVQTLVGGT-RFKEDQKRLEC-DPCQIIVATPG 474
SlDEAD13—101245343 ELAIQTHAVAKDLLKY--HS-------Q-TLGLVIGGS-ARRAEAE-RIA-KGANLLVGTPG 210
SlDEAD24—101249838 ELAGQLFEVLKSVGKH--QG-------F-SAGLLIGGR-KDVDAEK-EHV-NGLNILVCTPG 199
* * . ::: **.

```
                          motif III                    motif I
SlDEAD17—101264928        ALLNYLYSIDPERRRRSEFIRSVKYVVFDEADMLLC-GSFQNQVVRLIN--MLRFDEKQ   257
SlDEAD7—101255555         CIQTCLSNGVIQ---GKAVQDSLSILILDEADLLLS-YGYEDDLKALTS--HVP-----   197
SlDEAD39—101257152        RVLQHIEEGN-------MVYGDIRYLVLDEADTMFD-RGFGPDIRKFLA--PLKNRASK   296
SlDEAD29—101265585        RLWELMSGGETH---L-VELHSLSFFVLDEADRMIE-NGHFHELQSIVD--MLPMANKS   369
SlDEAD8—101259209         RLYDIMERLDI------LDFRDLEILILDEADRLLG-MGFEKQLNSIIS--RLP-----   194
SlDEAD32—101246967        RLMHHLSEVDD------MSLRTVEYVVFDEADCLFS-MGFAEQLHRILT--HLG-----   198
SlDEAD15—101266117        RMIDHLRNSMS------VDLDDLAVLILDEADRLLE-LGFSAEIRELVR--LCP-----   316
SlDEAD30—101259679        RIKVLIEQNPDI---P-PVFSKTKFLVLDEADRVLD-VGFEEELRAIFQ--CLP-----   226
SlDEAD31—101257016        RLLDHLSNTKG------FSLRTIKYLVLDEADRLLN-EDFEKALDQILN--AIP-----   181
SlDEAD14—101259928        RLKDMLAK-------KKMNLDNCRYLTLDEADRLVD-LGFEDDIREVFD--HF------   334
SlDEAD20—101260827        RLKDLLAK-------KKMNLDNCRYLTLDEADRLVD-LGFEDDIREVFD--HF------   334
SlDEAD6—101245423         RLLDCLER-------RYCVLNQCNYVVLDEADRMID-MGFGPQVVGVLD--AMPSSNMK   426
SlDEAD9—101267079         RLLDCLER-------RYCVLNQCNYVVLDEADRMID-MGFEPQVVGVLD--AMPSSNMK   475
SlDEAD1—101244218         RLVDLLER-------ARVSLQMIRYLALDEADRMLD-MGFEPQIRKIVQ--QMDM---P   326
SlDEAD11—101263192        RLVDLLER-------AKVSLQMIRYLALDEADRMLD-MGFEPQIRRIVQ--QMDM---P   336
SlDEAD28—101258585        RLIDHLQQ-------GNTSLGRIAFVVLDEADRMLD-MGFEPQIREVMR--NL------   290
SlDEAD27—101253217        RLQDLMEM-------GACNLKEVSFVVLDEADRMLD-LGFEPAVRAILS--QT------   309
SlDEAD2—101266555         RLNDILEM-------KRIDFRQVSLLVLDEADRMLD-MGFEPQIRKIVN--EI------   748
SlDEAD10—01254752         RLNDILEM-------RRVRLDQVSYLVLDEADRMLD-MGFEPQIRKIVK--EV------   327
SlDEAD36—101248651        RLNDILEM-------RRLSLGQVSYLVLDEADRMLD-MGFEPQIRKIVK--EV------   320
SlDEAD42—101254580        RMIDILCTSGG----KITNLRRVTYLVMDEADRMFD-MGFEPQITRIVQ--NT------   679
SlDEAD3—101264757         RLIDMIRK-------KAFTMLRATYLVLDEADRMFD-LGFEPQIRSIVG--QI------   404
SlDEAD12—101265421        RLIDMLEA-------QHTNLKRVTYLVLDEADRMLD-MGFEPQIRKLIS--QI------   405
SlDEAD37—544216           RLIDMLES-------NHTNLRRVTYLVLDEADRMLD-MGFEPQIRKIVS--QI------   271
SlDEAD16—101243705        TINKWVTG-------RKLGMSCMKILVFDEADHMLAESGFQDDSIRIMKAIVKA-----   270
SlDEAD34—101252541        TINKWVTA-------RKLGMSCMKILVFDEADHMLAESGFQDDSIRIMKAIVKA-----   278
SlDEAD25—101263737        RLIDLINN-------NTLKLGEVEYLVLDEADQMLA-VGFEEDVEVILE--KL------   282
SlDEAD23—101249660        RIIDLLKR-------GALNLAEIQFVVLDEADQMLN-VGFAEDVETILE--NV------   271
SlDEAD35—101259710        RIIDLLKR-------GSLNLSDIQFVVLDEADQMLN-VGFAEDVETILE--NI------   285
SlDEAD21—104648456        RVFDMLRR-------QSLRADHIKMFVLDEADEMLS-RGFKDQIYDIFQ--LL------   210
SlDEAD19—101244494        RVFDMLRR-------QSLRPDCLRMFVLDEADEMLS-RGFKDQIYDIFQ--ML------   210
SlDEAD40—101266108        RVFDMLRR-------QSLRPDHIKMFVLDEADEMLS-RGFKDQIYDIFQ--LL------   210
SlDEAD26—101252510        RVFDMLRR-------QSLRPDHIKMFVLDEADEMLS-RGFKDQIYDIFQ--LL------   210
SlDEAD41—101266405        RVFDMLRR-------QSLRPDHIKMFVLDEADEMLS-RGFKDQIYDIFQ--LL------   191
SlDEAD18—101258495        RILDLARK-------GVCVLKDCSMLVMDEADKMLS-PEFLPSIEQLIR--FL------   333
SlDEAD4—101253227         RILDLAKK-------GVCVLKDCSMFIMDEADKLLS-PEFQPSIEQLIC--FL------   294
SlDEAD33—101251630        RILDLARK-------GICVLKDCSMLVMDEADKLLS-PEFQPSIVQLIR--FL------   287
SlDEAD22—101245966        RLLDHLKNTSS----FLHT--NLRWIIFDEADRILE-LGYGKEIEDILN--ILGSKQQK   212
SlDEAD5—101245996         RLRDHVENTAG----FATRLMGVKVLVLDEADHLLD-MGFRKEIERIIS--AIP-----   586
SlDEAD38—101259722        RLLDHIENKSG----FSTRIMGLKMLILDEADHLLD-LGFRKDIEKLVD--CLP-----   521
SlDEAD13—101245343        RLLDHLRNTKG----FNYK--NLQCLVIDEADRILE-ANFEEDMQQILK--LLP-----   255
SlDEAD24—101249838        RLLQHMDETPN----FDCS--QLQVLVLDEADRILD-VGFKRDLNAIIS--QLP-----   244
                           :                        .:****:.   .      .
```

motif Ia

```
SlDEAD17—101264928   LKRRDWRRVRKIYERSKQYIFVAATLPENGKRTAGGVLK-R-------------------   357
SlDEAD7—101255555    --------------RRCQCLLMSATSSSDVEKLKKLILH-NPYILT----LPEVG-----   233
SlDEAD39—101257152   --------------EGFQTVLVTATMTKAVQKLVDEEFQ-GIEHLR----TSSLH-----   335
SlDEAD29—101265585   -------TVSSVQRKKRQTFVFSATIALSADFRKKL-KR-GSQKSK----ANDELNSIET   428
SlDEAD8—101259209    --------------KLRRTGLFSATQTEAVEELSKAGLR-NPVRVE----VRAEAKQ---   232
SlDEAD32—101246967   --------------ENRQTLLFSATLPSALAEFAKAGLR-DPQLVR----LDLDT-----   234
SlDEAD15—101266117   --------------KRRQTMLFSATMTEEVDELINLSLN-KPLRLS----ADPST-----   352
SlDEAD30—101259679   --------------KNRQTLLFSATMTSNLQTLLELSAN-KAYFYE----AYEGF-----   262
SlDEAD31—101257016   --------------HERRTYLFSATMTKKVRKLQRACLR-NPVKIE----AASKY-----   217
SlDEAD14—101259928   -------------KAQRQTLLFSATMPTKIQNFARSALV-KPITVN----VGRA------   370
SlDEAD20—101260827   -------------KAQRQTLLFSATMPTKIQNFARNALV-KPVIVN----VGRA------   370
SlDEAD6—101245423    -------K-LDENKIYRTTYMFSATMPPAVERLARNYLR-NPAVVT----IGTA------   473
SlDEAD9—101267079    -------E-LDEKRIYRTTYMFSATMPPAVERLARKYLR-NPVVVT----IGTA------   522
SlDEAD1—101244218    ------------PPGVRQTMLFSATFPKEIQRLASDFLS-SYIFLA----VGRV------   363
SlDEAD11—101263192   ------------PPGERQTMLFSATFPKEIQRLASDFLA-NYIFLA----VGRV------   373
SlDEAD28—101258585   -------------PVKHQTLLFSATMPAEIEALAQDYLT-NPVRIK----VGKV------   326
SlDEAD27—101253217   -------------CSVRQMVMFSATWPPEVHQLAQEFMDPNPIKVV----VGSE------   346
SlDEAD2—101266555    -------------PPQRQTLMYTATWPKEVRKIAGDLLR-NPVQVN----IGNV------   784
SlDEAD10—01254752    -------------PTRRQTLMYTATWPKEVRRIAADLLV-NPVQVN----IGNV------   363
SlDEAD36—101248651   -------------PKQRQTLMYTATWPKGVRKIAADLLV-NSVQVN----IGNV------   356
SlDEAD42—101254580   -------------RPDRQTVLFSATFPRQVEILARKVLN-KPVEIQ----VGGR------   715
SlDEAD3—101264757    -------------RPDRQTLLFSATMPRKVEKLAREILT-DPVRVT----VGEI------   440
SlDEAD12—101265421   -------------RPDRQTLYWSATWPREVEALARQFLR-NPYKVI----IGSP------   441
SlDEAD37—544216      -------------RPDRQTLYWSATWPD--------------------------------   286
SlDEAD16—101243705   -------------SANCQVLLFSATFGENVKAFVTKIVQ-DLFVQDYNQMFVKK------   310
SlDEAD34—101252541   -------------SANCQVLLFSATFGENVKAFVTKIVQ-DLFVQDYNQMFVKK------   318
SlDEAD25—101263737   -------------PPQRQSMLFSATMPGWVKKLSRKYLN-NPLTID---LVGDQ------   319
SlDEAD23—101249660   -------------PQKHQTLMFSATMPSWILKITNKFLK-NPVHID---LVGDS------   308
SlDEAD35—101259710   -------------RQKHQTMMFSATMPSWILKLTKKFLK-KPIHVD---LVGDS------   322
SlDEAD21—104648456   -------------PPKIQVGVFSATMPPEALEITRKFMN-KPVRIL---VKR--------   245
SlDEAD19—101244494   -------------PTKVQVGVFSATMPPEALDITRKFMN-KPVRIL---VKR--------   245
SlDEAD40—101266108   -------------PPKIQVGVFSATMPPEALEITRKFMN-KPVRIL---VKR--------   245
SlDEAD26—101252510   -------------PPKIQVGVFSATMPPEALEITRKFMN-KPVRIL---VKR--------   245
SlDEAD41—101266405   -------------PPKIQVGVFSATMPPEALEITRKFMN-KPVRIL---VKR--------   226
SlDEAD18—101258495   -------------PASRQILMFSATFPVTVKAFKDRYLQ-KPYVIN----LM--------   367
SlDEAD4—101253227    -------------PANRQILMFSATFPVTVKDFKERYLH-KPYVIN----LM--------   328
SlDEAD33—101251630   -------------PANRQVLMFSATFPVTVKDFKERYLQ-KPYVIN----LM--------   321
SlDEAD22—101245966   -------TTSQISEVQRQNVLISATLNEKVNHLAEISLDN-PVMVG----LDKKIELQLT   266
SlDEAD5—101245996    -------------KQ-RQTLLFSATVPPEVRQICHIALKRDHEFIN----TVEE------   622
SlDEAD38—101259722   -------------RR-RQSLLFSATVPKEVRRISQLVLKREYDYVD----TVGL------   557
SlDEAD13—101245343   -------------KEGRQTALFSATQTKKVEDLARLSLTA-PIYID----VDDG------   291
SlDEAD24—101249838   -------------KH-RQTLLFSATQTKSVQDLARLSLKD-PEYLG----VHEE------   279
                                           :**
```

motif Ib

```
SlDEAD17—101264928   -SMADSSPGVLRTMVFANTVDAVEAVANILTRV——G——————————————LECFR   439
SlDEAD7—101255555    ——LE—LVQKKVLIFTNSIDTSFRLKLFFEQF——G——————————————IKSAV   299
SlDEAD39—101257152   ——EPSLAKGNRVMVFCNTLNSSRAVDHFLNET——Q——————————————ISTVN   398
SlDEAD29—101265585   ——VHG——QGRTIVFCTSIAALRHISSLLRIL——G——————————————VNVWT   508
SlDEAD8—101259209    ——KN——KSKKIIVYFMTCACVDYWGTVLPRLSCLKS——————————————FSLIS   308
SlDEAD32—101246967   ——EQI-TSDQQTIVFVSTKYHVEFLNILLREE——G——————————————IEASV   296
SlDEAD15—101266117   ——LCTKTFTSKVIVFSGTKQAAHRLKIIFGLL——G——————————————FKAAE   415
SlDEAD30—101259679   ——KIKDIDVRSAIIFVSTCRSCQLLGLLLEEL——E——————————————IDAAA   325
SlDEAD31—101257016   ——EMS——GSTSMVFTRTCDATRLLALMLRNL——G——————————————LRAIP   277
SlDEAD14—101259928   ————————PPPVLVFCENKADVDDIHEYLLLK——G——————————————VEAVA   430
SlDEAD20—101260827   ————————PPPVLVFCENKADVDDIHEYLLLK——G——————————————VEAVA   430
SlDEAD6—101245423    ————————DKTAIVFINSRKRVNTVAKHLDKA——G——————————————YRVTT   534
SlDEAD9—101267079    ————————DKTAIVFINTKKQADFVAKNLDKN——G——————————————YRVTT   583
SlDEAD1—101244218    ENGV—HGKQALTLVFVETKKGADALEHWLCMN——G——————————————FPAAA   431
SlDEAD11—101263192   ANGV—HGKHSLTLVFVETKKGADALEHWLCIN——G——————————————FPATA   441
SlDEAD28—101258585   AQAEKSGHPFPLTIVFVERKTKCDEVAEALTQQ——G——————————————LLATA   396
SlDEAD27—101253217   K——————SRRNRVLVFVLYKKEASRVEIMLQKR——G——————————————WKVVS   411
SlDEAD2—101266555    ———————RGSKAIIFCSTKKLCDQLARSIG-R——N——————————————FGAAA   847
SlDEAD10—01254752    ———————PGSKIIIFCSTKKMCDQLARNLT-H——P——————————————FGAAA   426
SlDEAD36—101248651   ———————PGSKIIIFCSTKKMCDQLSRNLT-R——N——————————————FGAAA   419
SlDEAD42—101254580   ———————EKGKILIFVHTQEKCDALFKDLLKH——G——————————————YPCLS   777
SlDEAD3—101264757    ———————DNGDVLVFASKKATVDEIESQLAQK——G——————————————FRVAA   503
SlDEAD12—101265421   ———————DGSRILIFVETKKGCDQVTRQLRMD——G——————————————WPALS   504
SlDEAD37—544216      ———————DGSRILIFMDTKKGCDQVTRQLRMD——G——————————————WPALS   348
SlDEAD16—101243705   ——————QKVGQTIIFVRTRNSASMLHKSLVDY——G——————————————YEVTT   375
SlDEAD34—101252541   ——————QKVGQTIIFVRTRNSASMLHKSLVDY——G——————————————YEVTT   383
SlDEAD25—101263737   ——————AKGGKTIVFTQTKRDADEVSMAL-SN——S——————————————ISSEA   381
SlDEAD23—101249660   ——————AKGGKCIVFTQTKRDADKLSYVM-QK——S——————————————FNCEA   370
SlDEAD35—101259710   ——————AKGGKCIVFTQTKRDADRLAGAM-QR——T——————————————LRCEA   384
SlDEAD21—104648456   ——————AI-TQSVIFVNTRRKVDLLTEQMQSK——D——————————————HTVSA   308
SlDEAD19—101244494   ——————AI-TQSVIFVNTRRKVDWLTDKMRSR——D——————————————HTVSA   308
SlDEAD40—101266108   ——————AI-TQSVIFVNTRRKVDWLTDKMRGR——D——————————————HTVSA   308
SlDEAD26—101252510   ——————AI-TQSVIFVNTRRKVDWLTDKMRSR——D——————————————HTVSA   308
SlDEAD41—101266405   ——————AI-TQSVIFVNTRRKVDWLTDKMRGR——D——————————————HTVSA   289
SlDEAD18—101258495   ——————QI-NQSIIFCNSVNRVELLAKKITEL——G——————————————YSCFY   429
SlDEAD4—101253227    ——————QI-NQSIIFCNSVNRVELLAKKITEL——G——————————————YSCFY   390
SlDEAD33—101251630   ——————QI-NQSIIFCNSVNRVELLAKKITEL——G——————————————YSCFY   383
SlDEAD22—101245966   EK————EPSQKVVVFFSTCDAVDFHYSLVSGF——QWLSRQQSDTDVKQLFLKCNTFR   376
SlDEAD5—101245996    AD————DVNYKVLVFCTTAMVTKLVAELLGEL——N——————————————LNVRE   689
SlDEAD38—101259722   SE————VPDYKVIVFCTTAMMTSLMFSLFHEM——K——————————————MNVRE   623
SlDEAD13—101245343   S———————KKIMVFFSSCNSVKFHSELLRYI——K——————————————IECHD   354
SlDEAD24—101249838   N———————SRILVFLSSCKQVKFVFETFKKL——R—————————————PGIPLKC   344
                                 :::                .
```

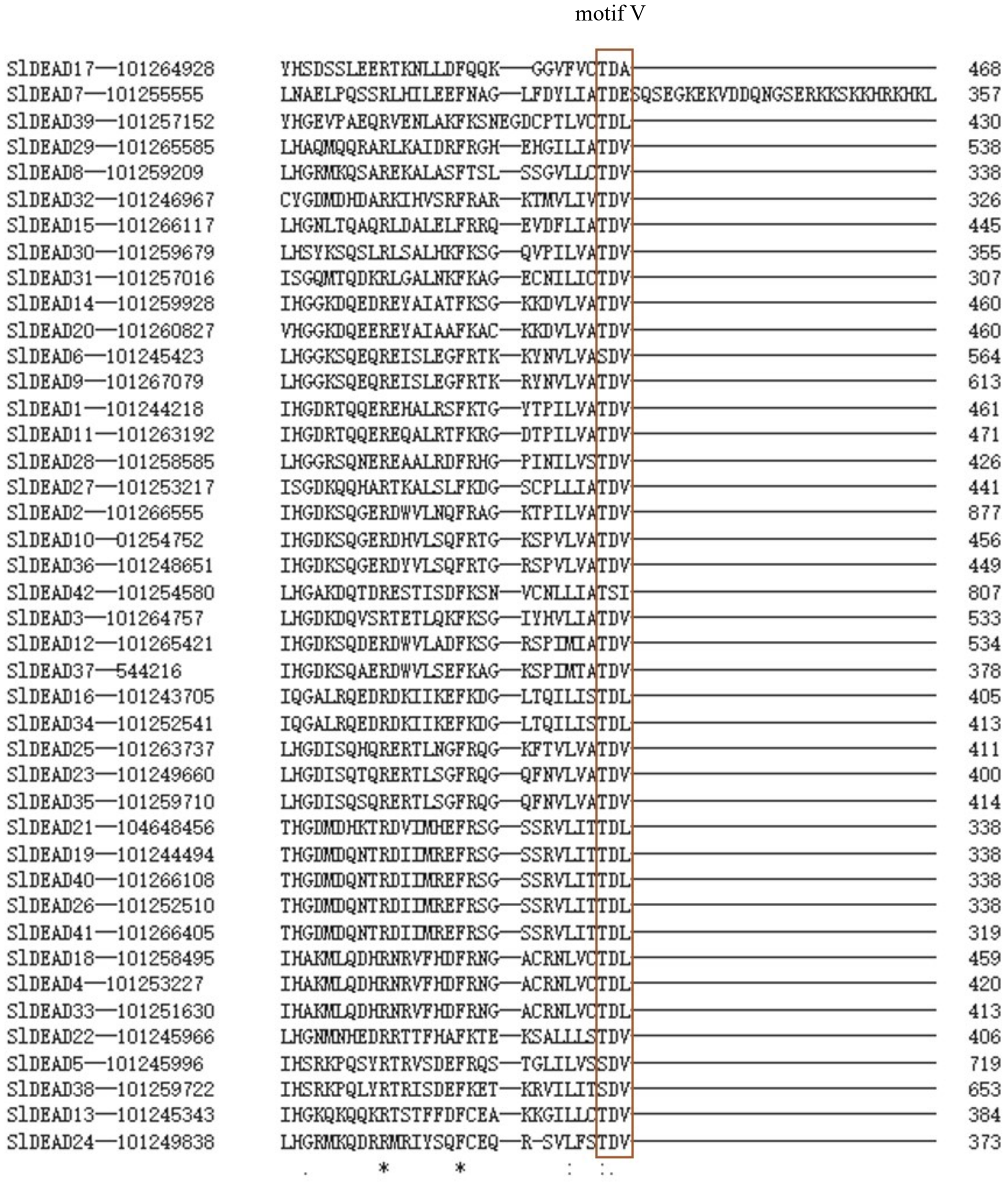
motif V
SlDEAD17—101264928 YHSDSSLEERTKNLLDFQQK--GGVFVCTDA 468
SlDEAD7—101255555 LNAELPQSSRLHILEEFNAG-LFDYLIATDESQSEGKEKVDDQNGSERKKSKKHRKHKL 357
SlDEAD39—101257152 YHGEVPAEQRVENLAKFKSNEGDCPTLVCTDL 430
SlDEAD29—101265585 LHAQMQQRARLKAIDRFRGH-EHGILIATDV 538
SlDEAD8—101259209 LHGRMKQSAREKALASFTSL-SSGVLLCTDV 338
SlDEAD32—101246967 CYGDMDHDARKIHVSRFRAR-KTMVLIVTDV 326
SlDEAD15—101266117 LHGNLTQAQRLDALELFRRQ-EVDFLIATDV 445
SlDEAD30—101259679 LHSYKSQSLRLSALHKFKSG-QVPILVATDV 355
SlDEAD31—101257016 ISGQMTQDKRLGALNKFKAG-ECNILICTDV 307
SlDEAD14—101259928 IHGGKDQEDREYAIATFKSG-KKDVLVATDV 460
SlDEAD20—101260827 VHGGKDQEEREYAIAAFKAC-KKDVLVATDV 460
SlDEAD6—101245423 LHGGKSQEQREISLEGFRTK-KYNVLVASDV 564
SlDEAD9—101267079 LHGGKSQEQREISLEGFRTK-RYNVLVATDV 613
SlDEAD1—101244218 IHGDRTQQEREHALRSFKTG-YTPILVATDV 461
SlDEAD11—101263192 IHGDRTQQEREQALRTFKRG-DTPILVATDV 471
SlDEAD28—101258585 LHGGRSQNEREAALRDFRHG-PINILVSTDV 426
SlDEAD27—101253217 ISGDKQQHARTKALSLFKDG-SCPLLIATDV 441
SlDEAD2—101266555 IHGDKSQGERDWVLNQFRAG-KTPILVATDV 877
SlDEAD10—01254752 IHGDKSQGERDHVLSQFRTG-KSPVLVATDV 456
SlDEAD36—101248651 IHGDKSQGERDYVLSQFRTG-RSPVLVATDV 449
SlDEAD42—101254580 LHGAKDQTDRESTISDFKSN-VCNLLIATSI 807
SlDEAD3—101264757 LHGDKDQVSRTETLQKFKSG-IYHVLIATDV 533
SlDEAD12—101265421 IHGDKSQDERDWVLADFKSG-RSPIMIATDV 534
SlDEAD37—544216 IHGDKSQAERDWVLSEFKAG-KSPIMTATDV 378
SlDEAD16—101243705 IQGALRQEDRDKIIKEFKDG-LTQILISTDL 405
SlDEAD34—101252541 IQGALRQEDRDKIIKEFKDG-LTQILISTDL 413
SlDEAD25—101263737 LHGDISQHQRERTLNGFRQG-KFTVLVATDV 411
SlDEAD23—101249660 LHGDISQTQRERTLSGFRQG-QFNVLVATDV 400
SlDEAD35—101259710 LHGDISQSQRERTLSGFRQG-QFNVLVATDV 414
SlDEAD21—104648456 THGDMDHKTRDVIMHEFRSG-SSRVLITTDL 338
SlDEAD19—101244494 THGDMDQNTRDIIMREFRSG-SSRVLITTDL 338
SlDEAD40—101266108 THGDMDQNTRDIIMREFRSG-SSRVLITTDL 338
SlDEAD26—101252510 THGDMDQNTRDIIMREFRSG-SSRVLITTDL 338
SlDEAD41—101266405 THGDMDQNTRDIIMREFRSG-SSRVLITTDL 319
SlDEAD18—101258495 IHAKMLQDHRNRVFHDFRNG-ACRNLVCTDL 459
SlDEAD4—101253227 IHAKMLQDHRNRVFHDFRNG-ACRNLVCTDL 420
SlDEAD33—101251630 IHAKMLQDHRNRVFHDFRNG-ACRNLVCTDL 413
SlDEAD22—101245966 LHGNMNHEDRRTTFHAFKTE-KSALLLSTDV 406
SlDEAD5—101245996 IHSRKPQSYRTRVSDEFRQS-TGLILVSSDV 719
SlDEAD38—101259722 IHSRKPQLYRTRISDEFKET-KRVILITSDV 653
SlDEAD13—101245343 IHGKQKQQKRTSTFFDFCEA-KKGILLCTDV 384
SlDEAD24—101249838 LHGRMKQDRRMRIYSQFCEQ-R-SVLFSTDV 373
. * * : :.

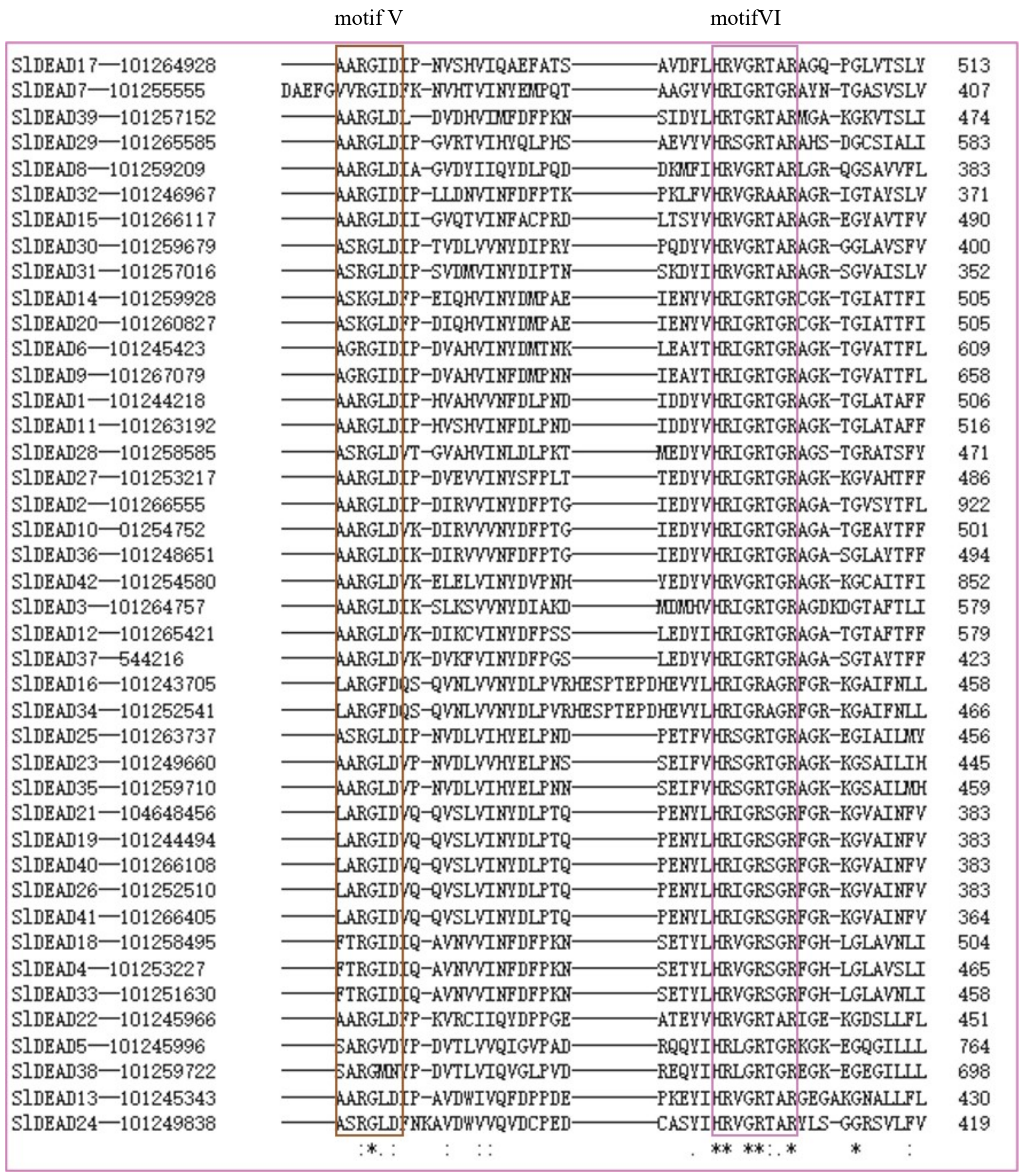
motif V
motifVI
SlDEAD17—101264928 ——AARGIDIP-NVSHVIQAEFATS——AVDFLHRVGRTARAGQ-PGLVTSLY 513
SlDEAD7—101255555 DAEFGVVRGIDFK-NVHTVINYEMPQT——AAGYVHRIGRTGRAYN-TGASVSLV 407
SlDEAD39—101257152 ——AARGLDL—DVDHVIMFDFPKN——SIDYLHRTGRTARMGA-KGKVTSLI 474
SlDEAD29—101265585 ——AARGLDIP-GVRTVIHYQLPHS——AEVYVHRSGRTARAHS-DGCSIALI 583
SlDEAD8—101259209 ——AARGLDIA-GVDYIIQYDLPQD——DKMFIHRVGRTARLGR-QGSAVVFL 383
SlDEAD32—101246967 ——AARGIDIP-LLDNVINFDFPTK——PKLFVHRVGRAARAGR-IGTAYSLV 371
SlDEAD15—101266117 ——AARGLDII-GVQTVINFACPRD——LTSYVHRVGRTARAGR-EGYAVTFV 490
SlDEAD30—101259679 ——ASRGLDIP-TVDLVVNYDIPRY——PQDYVHRVGRTARAGR-GGLAVSFV 400
SlDEAD31—101257016 ——ASRGLDIP-SVDMVINYDIPTN——SKDYIHRVGRTARAGR-SGVAISLV 352
SlDEAD14—101259928 ——ASKGLDFP-EIQHVINYDMPAE——IENYVHRIGRTGRCGK-TGIATTFI 505
SlDEAD20—101260827 ——ASKGLDFP-DIQHVINYDMPAE——IENYVHRIGRTGRCGK-TGIATTFI 505
SlDEAD6—101245423 ——AGRGIDIP-DVAHVINYDMTNK——LEAYTHRIGRTGRAGK-TGVATTFL 609
SlDEAD9—101267079 ——AGRGIDIP-DVAHVINFDMPNN——IEAYTHRIGRTGRAGK-TGVATTFL 658
SlDEAD1—101244218 ——AARGLDIP-HVAHVVNFDLPND——IDDYVHRIGRTGRAGK-TGLATAFF 506
SlDEAD11—101263192 ——AARGLDIP-HVSHVINFDLPND——IDDYVHRIGRTGRAGK-TGLATAFF 516
SlDEAD28—101258585 ——ASRGLDVT-GVAHVINLDLPKT——MEDYVHRIGRTGRAGS-TGRATSFY 471
SlDEAD27—101253217 ——AARGLDIP-DVEVVINYSFPLT——TEDYVHRIGRTGRAGK-KGVAHTFF 486
SlDEAD2—101266555 ——AARGLDIP-DIRVVINYDFPTG——IEDYVHRIGRTGRAGA-TGVSYTFL 922
SlDEAD10—01254752 ——AARGLDVK-DIRVVVNYDFPTG——IEDYVHRIGRTGRAGA-TGEAYTFF 501
SlDEAD36—101248651 ——AARGLDIK-DIRVVVNFDFPTG——IEDYVHRIGRTGRAGA-SGLAYTFF 494
SlDEAD42—101254580 ——AARGLDVK-ELELVINYDVPNH——YEDYVHRVGRTGRAGK-KGCAITFI 852
SlDEAD3—101264757 ——AARGLDIK-SLKSVVNYDIAKD——MDMHVHRIGRTGRAGDKDGTAFTLI 579
SlDEAD12—101265421 ——AARGLDVK-DIKCVINYDFPSS——LEDYIHRIGRTGRAGA-TGTAFTFF 579
SlDEAD37—544216 ——AARGLDVK-DVKFVINYDFPGS——LEDYVHRIGRTGRAGA-SGTAYTFF 423
SlDEAD16—101243705 ——LARGFDQS-QVNLVVNYDLPVRHESPTEPDHEVYLHRIGRAGRFGR-KGAIFNLL 458
SlDEAD34—101252541 ——LARGFDQS-QVNLVVNYDLPVRHESPTEPDHEVYLHRIGRAGRFGR-KGAIFNLL 466
SlDEAD25—101263737 ——ASRGLDIP-NVDLVIHYELPND——PETFVHRSGRTGRAGK-EGIAILMY 456
SlDEAD23—101249660 ——AARGLDVP-NVDLVVHYELPNS——SEIFVHRSGRTGRAGK-KGSAILIH 445
SlDEAD35—101259710 ——AARGLDVP-NVDLVIHYELPNN——SEIFVHRSGRTGRAGK-KGSAILMH 459
SlDEAD21—104648456 ——LARGIDVQ-QVSLVINYDLPTQ——PENYLHRIGRSGRFGR-KGVAINFV 383
SlDEAD19—101244494 ——LARGIDVQ-QVSLVINYDLPTQ——PENYLHRIGRSGRFGR-KGVAINFV 383
SlDEAD40—101266108 ——LARGIDVQ-QVSLVINYDLPTQ——PENYLHRIGRSGRFGR-KGVAINFV 383
SlDEAD26—101252510 ——LARGIDVQ-QVSLVINYDLPTQ——PENYLHRIGRSGRFGR-KGVAINFV 383
SlDEAD41—101266405 ——LARGIDVQ-QVSLVINYDLPTQ——PENYLHRIGRSGRFGR-KGVAINFV 364
SlDEAD18—101258495 ——FTRGIDIQ-AVNVVINFDFPKN——SETYLHRVGRSGRFGH-LGLAVNLI 504
SlDEAD4—101253227 ——FTRGIDIQ-AVNVVINFDFPKN——SETYLHRVGRSGRFGH-LGLAVSLI 465
SlDEAD33—101251630 ——FTRGIDIQ-AVNVVINFDFPKN——SETYLHRVGRSGRFGH-LGLAVNLI 458
SlDEAD22—101245966 ——AARGLDFP-KVRCIIQYDPPGE——ATEYVHRVGRTARIGE-KGDSLLFL 451
SlDEAD5—101245996 ——SARGVDYP-DVTLVVQIGVPAD——RQQYIHRLGRTGRKGK-EGQGILLL 764
SlDEAD38—101259722 ——SARGMNYP-DVTLVIQVGLPVD——REQYIHRLGRTGREGK-EGEGILLL 698
SlDEAD13—101245343 ——AARGLDIP-AVDWIVQFDPPDE——PKEYIHRVGRTARGEGAKGNALLFL 430
SlDEAD24—101249838 ——ASRGLDFNKAVDWVVQVDCPED——CASYIHRVGRTARYLS-GGRSVLFV 419
:*.: : :: . ** **:.* * :
